# Exosome-functionalized photocrosslinked GelMA/HAMA hydrogel promotes facial nerve recovery via inflammatory microenvironment regulation

**DOI:** 10.1016/j.bioactmat.2026.01.008

**Published:** 2026-01-19

**Authors:** Chun Chen, Yifei Zhang, Linchao Zhang, Israr Ullah, Lei Hang, Yupeng Liu, Jun Yang

**Affiliations:** a Department of Otorhinolaryngology-Head & Neck Surgery, Xinhua Hospital, Shanghai Jiao Tong University School of Medicine, Shanghai, 200092, China; b Ear Institute, Shanghai Jiao Tong University School of Medicine, Shanghai, 200092, China; c Shanghai Key Laboratory of Translational Medicine on Ear and Nose Diseases, Shanghai, 200092, China; d Business School, Shanghai Normal University Tianhua College, Shanghai, 201815, China; e Yangtze River Delta Research Institute, University of Electronic Science and Technology of China, Quzhou, China; f Department of Computer Science, Virtual University of Pakistan, Off Raiwind Road, Lahore, 54000, Pakistan

**Keywords:** Facial nerve injury, Nerve regeneration, Stem cells, Exosomes, Hydrogel, Macrophage polarization

## Abstract

Facial nerve crush injuries frequently lead to incomplete functional restoration owing to constrained regenerative approaches and suboptimal treatment methods. While hydrogel-based systems have emerged as viable alternatives among bioengineered scaffolds, their therapeutic potential remains compromised by inadequate biological activity and unfavorable inflammatory conditions. Our research engineered a photoactivated GelMA/HAMA composite hydrogel incorporating bone marrow mesenchymal stem cell-derived exosomes (BExos), with comprehensive characterization of its material attributes. We systematically assessed the biomaterial's regenerative capacity through in vitro experiments involving BMSCs and RAW264.7 macrophages, complemented by comprehensive in vivo evaluations in a rodent facial nerve injury model incorporating functional restoration metrics, neurophysiological testing, tissue analysis, and biomolecular profiling. The BExos-integrated hydrogel established a favorable niche promoting BMSCs transdifferentiation toward Schwann cell-mimetic lineages while demonstrating marked improvement in neuromuscular functional restoration. Compared to untreated cohorts, the composite hydrogel demonstrated enhanced axonal regrowth, improved remyelination processes, and notably reduced oxidative damage. The biomaterial effectively shifted macrophage differentiation from M1 pro-inflammatory states toward M2 anti-inflammatory phenotypes through modulation of PI3K/NF-κB/P38 signaling cascades, with *Neuronatin* emerging as a key regulatory element in this pathway. Mechanistic investigations demonstrated that the therapeutic benefits stemmed from synergistic structural reinforcement combined with exosome-mediated immune regulation, positioning this dual-action hydrogel as an innovative solution for facial nerve repair.

## Highlights

• A photocrosslinked GelMA/HAMA hydrogel loaded with BMSC exosomes was engineered as a biomimetic 3D niche for facial nerve repair.
• BExos@GelMA/HAMA hydrogel significantly promoted axonal regrowth, myelination and functional recovery in facial nerve injury model.
• Immunomodulatory microenvironment was regulated by shifting macrophages from M1 to M2 phenotype via PI3K/NF-κB/P38 pathways.
• *Nnat* exerts anti-inflammatory effects by inhibiting the PI3K/NF-κB/P38 signaling pathway, thereby promoting macrophage polarization to M2.

## Graphical abstract

**Figure:**
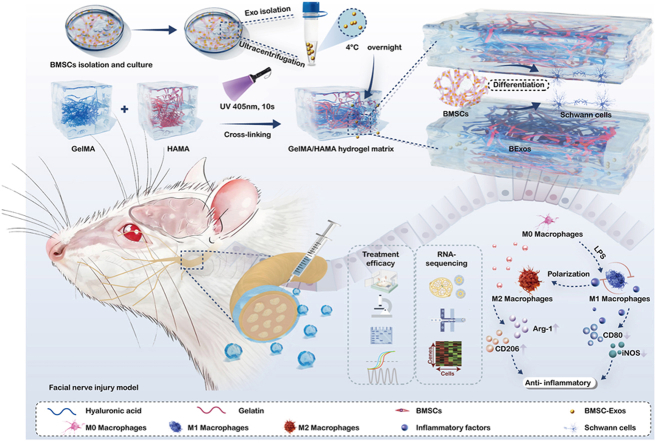

## 1 Introduction

The facial nerve represents one of the most extensive and externally positioned cranial nerves, characterized by remarkably elaborate anatomical pathways [1]. Peripheral facial paralysis (PFP), a frequently encountered neurological disorder, stands as the predominant manifestation of cranial motor nerve impairment, occurring in approximately 20–30 individuals per 100,000 globally annually [2]. Mechanical injury to the facial nerve initiates Wallerian degeneration processes manifested through axon fragmentation, demyelination phenomena, and concurrent inflammatory mechanisms [3]. This degenerative phase stimulates Schwann cell multiplication, culminating in Büngner band development that establishes directional pathways for controlled neural regrowth, effectively preventing aberrant connections between regenerating axons and disconnected muscle groups [4]. These specialized glial cells serve fundamental functions in neural restoration through axon ensheathment, myelin production, and secretion of growth-promoting molecules vital for nerve repair [5]. Following nerve damage, Schwann cells adopt a regenerative state that provides directional signals for axon extension [6]. Peripheral nerve repair involves three key mechanisms: myelin sheath reformation, axon elongation, and synaptic reorganization, where Schwann cells critically coordinate these processes to facilitate neural recovery [7]. Traumatic neural injury initiates rapid inflammatory reactions that attract macrophage infiltration to damaged areas [8]. These glial cells participate in myelin breakdown through metalloproteinase-mediated degradation of myelin basic protein (MBP) [2]. Immune cells including macrophages migrate through blood vessels to engulf myelin debris and damaged neural components, working synergistically with Schwann cells to remove post-axotomy waste — a fundamental requirement for proper nerve regrowth [9].Current regenerative approaches for peripheral nerve repair, including neurotrophic factor administration, exhibit restricted clinical effectiveness owing to cytokine instability from enzymatic breakdown after delivery and insufficient spatial precision in targeting injury zones. The inflammatory milieu surrounding neural lesions substantially determines regenerative success [10]. Central to this process lies macrophage polarization dynamics, where these immune cells demonstrate remarkable functional adaptability. Following neural trauma, pro-inflammatory M1 macrophages dominate initial responses, yet their prolonged activation may intensify structural damage, highlighting the critical importance of timely transition to reparative phenotypes [11]. Characterized by anti-inflammatory mediator secretion and regenerative niche formation, M2-polarized macrophages prove indispensable for inflammatory resolution and axonal regeneration [[12], [13], [14]]. However, current therapeutic strategies encounter significant obstacles in precisely controlling this phenotypic conversion process, particularly in achieving spatiotemporal coordination between immune modulation and neural repair mechanisms. Macrophage differentiation toward the regenerative M2 phenotype remains suboptimal, hindering the achievement of optimal regenerative outcomes. These biological limitations have propelled the development of engineered tissue solutions, particularly emphasizing scaffold-based technologies and cellular regeneration approaches [15]. Advanced biomaterial constructs combining structural frameworks with programmable therapeutic molecule delivery systems represent an innovative direction for neural restoration [16]. Such engineered matrices achieve dual functionality by mimicking native extracellular environments through controlled neurotrophic factor elution while establishing permissive microarchitectures for nerve fiber regrowth [17]. In cellular therapeutic strategies, mesenchymal stem cells isolated from bone marrow (BMSCs) have emerged as a leading candidate due to their potent secretory profile containing multiple neuroregulatory proteins [18]. However, practical application of BMSCs transplantation faces multiple biological barriers including host immune responses, poor engraftment efficiency, and the therapeutic potential of BMSC-derived exosomes (BExos) faces challenges due to suboptimal engraftment efficiency. These nanoscale vesicles have emerged as promising acellular alternatives, preserving the reparative properties of their progenitor cells while circumventing the risks linked to direct cell transplantation [19]. Containing diverse biological cargo such as regulatory proteins, lipid mediators, and genetic materials, BExos demonstrate capacity to orchestrate multiple neural repair mechanisms including immune regulation, vascular network formation, and neurite elongation [20]. Despite these advantages, clinical application continues to face challenges from their short biological half-life and absence of structural scaffolds necessary for prolonged neural tissue regeneration.

To address these limitations, hydrogel-based delivery systems have been developed to offer adjustable mechanical properties, biological compatibility, and extracellular matrix-mimicking capabilities. Emerging advancements highlight photo-responsive hydrogels as innovative neural support matrices, particularly gelatin methacryloyl (GelMA) and hyaluronic acid methacryloyl (HAMA) formulations which have gained substantial research attention [21]. These biologically sourced polymer networks promote cellular adhesion and migration while supporting proliferation through their three-dimensional architectures. The biodegradable nature and tissue compatibility enhance their suitability for clinical translation, establishing these materials as valuable resources for tissue regeneration strategies [22]. Research has confirmed that incorporating gelatin hydrogels with basic fibroblast growth factor (bFGF) into nerve conduits significantly enhances axonal extension and neural repair in sciatic nerve injury models [23]. However, gelatin's inherent structural instability and rapid proteolytic breakdown require blending with complementary biomaterials or implementing crosslinking modifications to optimize hydrogel functionality [24]. Hyaluronic acid (HA)-based hydrogels demonstrate exceptional fluid retention and controlled enzymatic breakdown, establishing hydrated microenvironments with interconnected porosity at lesion sites that effectively reduce glial scar progression [25]. While reactive gliosis initially provides neuroprotection through inhibitory molecule secretion, chronic scar maturation generates physical obstructions and sustained molecular inhibition that obstruct neuronal regeneration [26]. Furthermore, HA hydrogel matrices support enhanced cellular migration and angiogenesis development while maintaining structural integrity during tissue remodeling phases. Emerging research indicates that enzyme-crosslinked hyaluronic acid matrices accelerate neurite outgrowth while enhancing axonal and dendritic development in neural tissue engineering applications. These biomaterial constructs facilitate synaptic network formation and maintain functional electrophysiological activity in living systems when implemented for central nervous system repair strategies [27]. Recent advancements in bioengineered matrices reveal that embedding MSC-derived exosomes within GelMA/HAMA composites creates neuroregenerative microenvironments through dual mechanisms: providing structural support for neural differentiation while modulating inflammatory reactions through exosome-driven macrophage reprogramming and subsequent signaling cascade regulation [28].

**Fig. 1:**
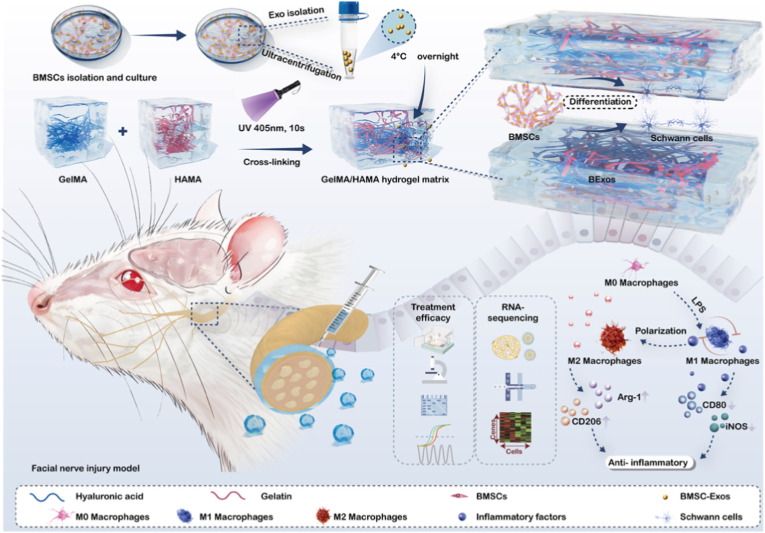
Schematic illustration of the BExos-encapsulated GelMA/HAMA hydrogel. The composite construct facilitates Schwann cell adhesion, proliferation, and axonal regeneration, while modulating macrophage polarization to induce a pro-regenerative microenvironment conducive to facial nerve repair.

This study hypothesizes that the BExos@GelMA/HAMA hydrogel composite synergistically enhances cranial nerve regeneration through dual mechanisms of axonal reconstruction and immune microenvironment regulation (Fig. 1). The engineered scaffold establishes a 3D extracellular matrix architecture that maintains mechanical stability while facilitating neural tissue restoration. Systematic in vitro and in vivo evaluations demonstrated the composite's optimal cytocompatibility, mitogenic potential for neural cells, and therapeutic performance in neurological recovery. However, the precise biomolecular mechanisms governing macrophage polarization regulation require further exploration. Through comprehensive transcriptome profiling, this research identified *Neuronatin* (*Nnat*) as a pivotal regulatory element in hydrogel-mediated immunomodulation. While *Nnat*'s established functions involve neurodevelopmental processes and calcium homeostasis, its role in regulating macrophage phenotypic transitions during neural repair warrants detailed mechanistic investigation. Regeneration processes have yet to be fully elucidated. Together, these engineered biomaterials create an immune-regulatory niche that supports cranial nerve repair, offering innovative strategies for advancing peripheral nerve regeneration and therapeutic interventions.

## 2 Results and discussion

### 2.1 Preparation and characterization of photocrosslinked GelMA/HAMA hydrogels

**Fig. 2:**
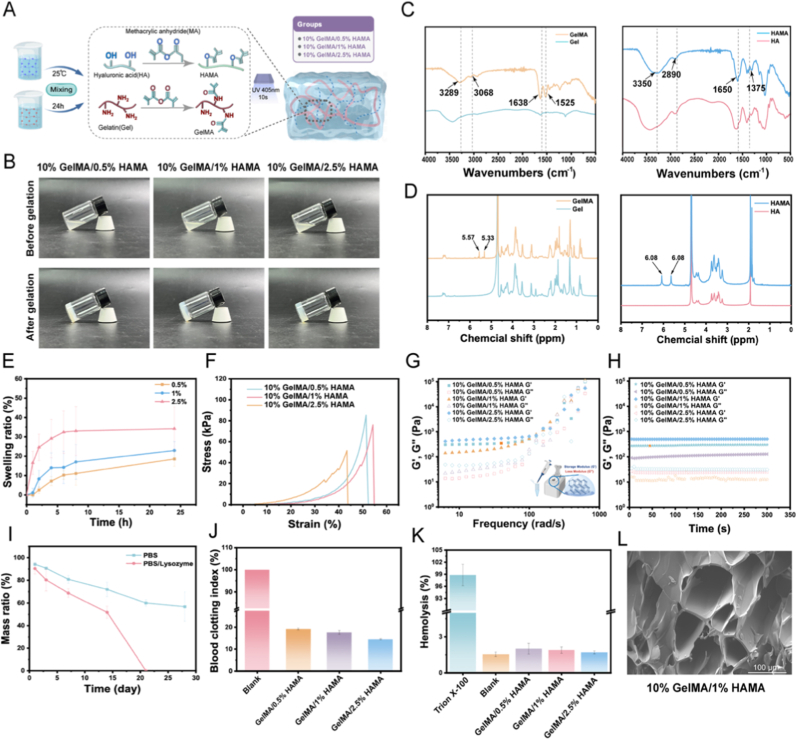
Preparation and characterization of photo-Crosslinked GelMA/HAMA hydrogels. (A) Schematic diagram of photo-Crosslinked GelMA/HAMA hydrogels. (B) Optical images of mixing GelMA and HAMA at varying ratios (10 % GelMA with 0.5 %, 1 %, or 2.5 % HAMA) composite scaffolds. (C) GelMA and HAMA NMR spectrum. (D) GelMA and HAMA infrared spectrum. (E) Swelling kinetics profiles of GelMA/HAMA hydrogels showing rapid initial uptake and equilibrium stabilization. (F) Compressive stress-strain curves of GelMA/HAMA hydrogels with varying HAMA concentrations. (G and H) Rheological characterization showed time and frequency-dependent storage (G′) and loss (G″) moduli. (I) Degradation curve of 10 % GelMA/1 % HAMA hydrogel in PBS and lysozyme environment. (J) Coagulation index was decreased in GelMA/HAMA hydrogels. (K) Hemolysis rates showed excellent blood compatibility. (L) SEM images of the cross-sectional morphology of 10 % GelMA/1 % HAMA. Scale bars:100 μm.

The methacrylation process for gelatin and hyaluronic acid was accomplished via dehydration-condensation interactions between methacrylic anhydride's reactive groups and biopolymer functional sites, generating polymer networks enriched with photo-responsive carbon-carbon double bonds. This hydrogel system integrates GelMA's structural advantages with HAMA's bioactive characteristics for neural regeneration applications. GelMA preserves collagen-derived RGD peptide sequences that promote cellular attachment through integrin receptor binding, enabling effective neuronal substrate interactions as documented in prior research [29]. Concurrently, HAMA contributes native hyaluronan signaling elements that engage cell-surface receptors like CD44 and RHAMM, influencing migratory behavior and inflammatory modulation critical for nerve repair processes [30]. Fabrication involved mixing varying concentrations of HAMA (0.5–2.5 %) with 10 % GelMA followed by UV-induced crosslinking (405 nm wavelength) using LAP photoinitiator, as illustrated in Fig. 2A. The 405 nm irradiation parameter was strategically chosen to balance polymerization efficiency with biological compatibility, minimizing potential cellular damage during network formation. To address premature dissolution issues in biological components, we implemented a novel 405 nm irradiation system as an alternative to conventional 365 nm UV exposure [31]. GelMA and HAMA powders were dissolved in LAP-containing phosphate-buffered saline (PBS) through lyophilization reconstitution, forming precursor solutions with excellent flow properties and colloidal stability. These hydrogel formulations demonstrated optical clarity ranging from transparent to light amber hues while maintaining uniform viscoelastic characteristics. Thermal dissolution testing at physiological temperature (37 °C) revealed complete liquefaction within 28–32 min, producing an injectable fluid with viscosity measuring 1.15–1.25 Pa s at standard conditions. Our optimized composition containing 10 % (w/v) GelMA and 1 % (w/v) HAMA in PBS with 0.5 % LAP photocured rapidly under 405 nm illumination, achieving 88–91 % crosslinking efficiency within 8–9 s of exposure. While increasing HAMA concentration to 2 % enhanced structural integrity, we observed compromised syringe ability due to elevated flow resistance from enhanced polymer entanglement [32]. The optimized 10 % GelMA/1 % HAMA composite demonstrated optimal balance in mechanical properties, crosslinking efficiency, and clinical applicability while preserving injectability. Consistent batch reproducibility exhibited minimal variability (<5 % coefficient of variation), ensuring manufacturing consistency suitable for facial nerve regeneration procedures (Fig. 2B). Structural characterization through nuclear magnetic resonance (NMR) and fourier-transform infrared spectroscopy (FT-IR) spectroscopy confirmed successful chemical modifications. NMR analysis of GelMA revealed characteristic vinyl proton resonances between 5.33 and 5.57 ppm, verifying methacrylation of lysine residues post-modification. Complementary FT-IR spectra showed distinctive absorption bands corresponding to amide bonds and methacrylate functional groups. The NMR analysis of HAMA revealed distinct resonance signals at 5.65 and 6.08 ppm, validating effective functionalization through methacrylate group incorporation (Fig. 2C). FT-IR characterization of GelMA displayed diagnostic absorption bands at 3289 cm^−1^ (hydroxyl and amine stretching), 1638 cm^−1^ (amide carbonyl vibration), and 1525 cm^−1^ (secondary amide deformation), confirming successful gelatin derivatization. HAMA's spectral profile exhibited broad vibrational modes between 3350 and 2976 cm^−1^ (hydroxyl/amine stretching) with characteristic absorptions at 2890 cm^−1^ (aliphatic C-H) and 1375 cm^−1^ (C-N stretching). The prominent 1650 cm^−1^ peak confirmed methacryloyl group attachment, while preserved spectral patterns indicated maintained hyaluronic acid backbone integrity (Fig. 2D). Quantitative analysis determined methacrylation efficiencies of 72 ± 3 % for GelMA and 58 ± 2 % for HAMA, achieving optimal balance between photocrosslinking capability (through available methacrylate groups) and biomolecular functionality preservation. The swelling behavior of GelMA/HAMA hydrogels was investigated in PBS at physiological temperature (37 °C) as shown in Fig. 2E. All hydrogels reached swelling equilibrium within 8 h and maintained stable swelling rates thereafter, indicating their excellent dimensional stability. Uniaxial compression tests indicated that the mechanical properties of GelMA/HAMA hydrogels are significantly influenced by HAMA concentration (Fig. 2F). The 10 % GelMA/2.5 %HAMA hydrogel exhibits a maximum compressive stress of 50 kPa, while the 10 % GelMA/0.5 %HAMA hydrogel reaches 85 kPa. These results demonstrate that the compressive stress of GelMA/HAMA hydrogels can be precisely controlled by adjusting HAMA concentration. This tunable compressive stress capability indicates the material's superior adaptability to diverse tissue and organ applications, making it a promising biomaterial with significant biomedical potential. Rheological analysis revealed that the GelMA/HAMA hydrogels predominantly exhibited elastic behavior, with storage modulus (G′) significantly exceeding the loss modulus (G″) across the measured frequency spectrum (Fig. 2G). Time-sweep evaluations showed minimal variation (<5 %) in G′ over 300 s of oscillatory testing, confirming the hydrogel's stability under dynamic mechanical conditions relevant to nerve repair. The non-intersection of G′ and G″ within the tested frequency range further corroborates the hydrogel's elastic dominance (Fig. 2H). Increasing HAMA content from 0.5 % to 2 % resulted in a linear rise in G′ (R^2^ = 0.97), with the 1 % HAMA formulation achieving an optimal balance of viscoelastic properties, aligning with the soft tissue modulus of 1–3 kPa typical of native nerve tissue and supporting Schwann cell viability and function [33]. Fig. 2I presents the degradation kinetics of the 10 % GelMA/1 % HAMA hydrogel in PBS supplemented with 10,000 U/mL lysozyme. The hydrogel demonstrated a markedly enhanced degradation rate, losing over 50 % of its mass within 14 days and achieving complete degradation by day 21. Conversely, under control PBS conditions lacking enzymatic activity, degradation proceeded gradually, underscoring the pivotal role of enzymatic catalysis in hydrogel matrix depolymerization [34]. This degradation process highlights the enzyme-sensitive characteristics of the GelMA/HAMA hydrogel, primarily due to the presence of matrix metalloproteinase (MMP)-responsive sequences within GelMA [35]. This bioresponsive degradation supports gradual scaffold replacement by regenerating neural tissue, minimizing secondary injury and surgical removal probability.

The hydrogels' minimal hemolytic activity and regulated pro-coagulant properties ensure biocompatibility with blood components and facilitate hemostasis. Coagulation testing revealed that the hydrogels significantly enhanced platelet adhesion and activation, reducing whole blood clotting time by 79 ± 4 % relative to controls (*p* < 0.001, Fig. 2J). This suggests that the hydrogels support the formation of a stable fibrin mesh, promoting surgical hemostasis without provoking pathological thrombosis [36]. All formulations demonstrated hemolysis rates below 3.0 % (Fig. 2K), well under the 5 % limit specified by ISO 10993-4 for blood-contacting materials, indicating that the hydrogel surfaces do not compromise red blood cell membrane integrity [37]. The low hemolytic response was unaffected by variations in HAMA concentrations (0.5–2.5 %), implying that the methacrylation process does not impart cytotoxicity. The fabricated hydrogels were lyophilized and examined via scanning electron microscopy (SEM). As depicted in Fig. 2L, all samples exhibited an interconnected porous architecture with pore sizes ranging from 50 to 200 μm. The smooth, microfibrous pore walls indicated successful polymer integration without phase separation. These porous structures provide ample surface area for cellular adhesion, proliferation, and differentiation, along with channels facilitating molecular and nutrient exchange. The pore size and high interconnectivity (85 ± 5 %) surpassed the 20 μm threshold necessary for Schwann cell infiltration and axonal guidance, while the fibrous morphology closely replicates native extracellular matrix components, supporting cellular integration with the scaffold [38]. Increasing the HAMA content to 2.5 % generated secondary nanopores (50–100 μm) (Fig. S1).

### 2.2 BExos-loaded hydrogel promoted BMSCs proliferation, activity and differentiation in vitro

**Fig. 3:**
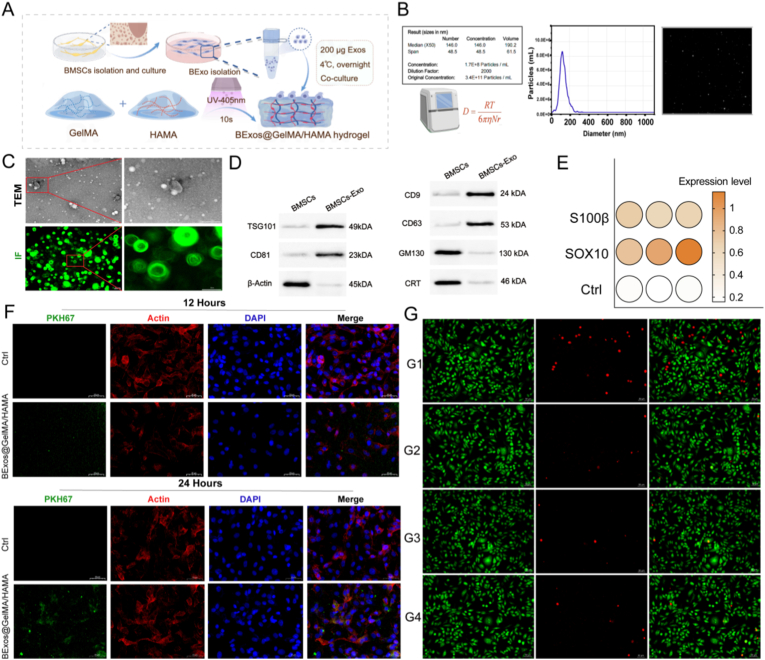
Impact of BExos@GelMA/HAMA hydrogel in vitro. (A) Synthesis flowchart of BExos@GelMA/HAMA hydrogel composite. (B) NTA showed size distribution of BMSC-derived exosomes. (C) Upper: Distribution of exosomes under TEM. Scale bars:100 nm. Down: 3D immunofluorescence staining of exosomes. Scale bars: 200 nm, 50 nm. (D) Western blot of specific exosomes markers CD81, TSG101, CD9, CD63, GM130, CRT. (E) S100β and SOX10 upregulation in hydrogel group. (F) Fluorescence micrographs showed spatiotemporal dynamics of exosome-mediated endocytosis on composite hydrogels. Scale bars: 50 μm. (G) Calcein-AM/PI staining showed enhanced cell viability in composite hydrogels. Scale bars: 50 μm. (n = 5, G1: Control. G2: BExos. G3: GelMA/HAMA. G4: BExos@GelMA/HAMA. ∗*p <* 0.05; ∗∗*p <* 0.01; ∗∗∗*p <* 0.001.)

Primary mesenchymal stromal cells (MSCs) from adult male Sprague-Dawley rats were isolated and expanded in vitro, followed by ultracentrifugation for exosome isolation. A bioactive exosome-laden GelMA/HAMA hydrogel composite was fabricated by homogeneously combining MSC-derived exosomes with a sterile 10 % GelMA/1 % HAMA precursor solution, which was photocrosslinked under 405 nm ultraviolet illumination for 10 s (Fig. 3A). Nanoparticle tracking analysis (NTA) revealed MSC-derived exosomes with a modal diameter of 146 ± 2.2 nm (Supplementary Video 1), consistent with the International Society for Extracellular Vesicles (ISEV) standard range of 30–200 nm. The high correlation coefficient (R^2^ > 0.98) in Brownian motion analysis confirmed measurement accuracy, while background noise remained below 10 particles per field of view, indicating high exosome purity [39]. Furthermore, the exosomes have a particle size of approximately 100 nm with a uniform distribution (Fig. 3B). Furthermore, we measured the potential of the exosomes, which was −30.1 mV, within the typical range of exosomes (−10 to −70 mV) (Fig. S2). Compared with adipose-derived mesenchymal stem cell (ADMSC) exosomes, BMSC-derived exosomes exhibit a lower particle size (Fig. S3). Transmission electron microscopy (TEM) and three-dimensional immunofluorescence imaging illustrated the characteristic cup-shaped morphology of isolated exosomes, with intact double membranes and diameters ranging from 80 to 160 nm (Fig. 3C), aligning with MISEV2018 guidelines [40]. Western blotting confirmed the presence of exosome markers CD81 (23 kDa), TSG101 (49 kDa), CD63 (53 kDa) (Fig. 3D). Furthermore, the negative markers GM130 (130 kDa) and CRT (46 kDa) were not detected in the exosomes (Fig. S4). These findings collectively affirm the successful isolation of high-purity MSC-derived exosomes suitable for incorporation into hydrogels for nerve regeneration therapies. Furthermore, we established an exosome release profile over 28 days and demonstrated that these bioactive exosomes were effectively encapsulated in the composite hydrogel, negligible immune cell infiltration facilitated sustained release of BMSCs-Exos beyond 28 days, with over 90 % of the exosomes being released during this period (Fig. S5).

Passage 2 (P2) MSCs were harvested and seeded at a density of 2-3 × 10^4^ cells per well onto two substrates: the MSC-derived exosome-loaded GelMA/HAMA hydrogel and conventional tissue culture plates (Fig. S6). Morphological analysis revealed that MSCs cultured on hydrogels underwent significant differentiation, exhibiting spindle-shaped or elliptical morphology with uniform adhesion by day 3. Cells oriented along the hydrogel's fibrillar matrix, forming networks reminiscent of native neural tissue (Fig. S7). The hydrogel's mechanical stiffness, ranging from 35 to 180 kPa, likely facilitated differentiation, resulting in cell phenotypes sharing approximately 85 % morphological similarity with primary Schwann cells [41]. To assess the ability of neuronal differentiation, q-PCR was conducted to measure the Schwann cell markers S100β and SOX10, which are key regulators of myelination and nerve regeneration [42]. The composite hydrogel group showed markedly higher expression of both genes compared with the control by bubble chart (Fig. 3E).

By day 7, cells exhibited elongated cytoplasmic extensions exceeding 100 μm, with a 2.6-fold increase in cell area and a 3.6-fold rise in aspect ratio relative to controls (*p* < 0.001), indicating Schwann-like differentiation. Absorbance measurements at this time point revealed significantly higher proliferation in the MSC exosome-loaded hydrogel group (1.39 ± 0.08) versus control (1.26 ± 0.05, *p* < 0.001, Fig. S7). Immunofluorescence imaging displayed rapid internalization of PKH67-labeled exosomes, with peri-nuclear accumulation observed within 12 h of seeding. After 48 h, approximately 78.4 ± 5.2 % of DAPI-stained nuclei exhibited exosome uptake, validating efficient internalization. Confocal images confirmed intracellular localization of PKH67-labeled exosomes concentrated around the nucleus (Fig. 3F). Live/dead assays, employing Calcein-AM and propidium iodide (PI), indicated high biocompatibility, with cell viability reaching 92.3 ± 3.1 % in the exosome-laden hydrogel group compared to 85.7 ± 2.8 % in controls after 7 days (*p* < 0.01). Cultured MSCs within the hydrogel displayed elongated, spindle-like morphology consistent with active proliferation (Fig. 3G).

### 2.3 Synergistic enhancement of facial nerve repair via BExos@GelMA/HAMA hydrogel loaded in crush injury model

**Fig. 4:**
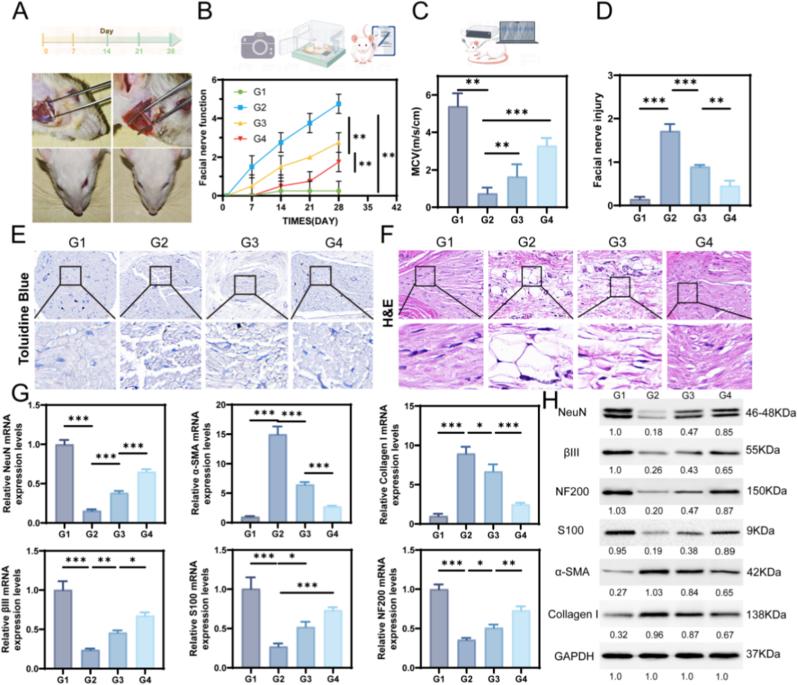
In vivo assessment of facial nerve repair and functional recovery. (A) Photos of nerve injury site and facial functional assessments at 0d and 28d in experimental group. (B) Comparison of the total scores of facial nerve function assessment. (C) EMG detection of nerve conduction velocity. (D,E,F) Staining results of H&E and TB. Scale bars: 50, 10 μm. (G) Quantification of facial nerve function-related gene levels. (H) The protein expressions of NeuN, βIII, NF200, S100, α-SMA, Collagen I. scale bars: 50 μm. (n = 5, G1: Sham. G2: PBS-treated. G3: GelMA/HAMA. G4: BExos@GelMA/HAMA. ∗*p <* 0.05; ∗∗*p <* 0.01; ∗∗∗*p <* 0.001.)

To evaluate the therapeutic effects on neural repair after facial nerve injury, researchers must consider the innate regenerative potential that might be overlooked in full-transection studies. Complete nerve severance models often result in aberrant axonal pathfinding and suboptimal functional recovery. Our experimental design prioritized functional reinnervation quality over mere axonal growth metrics, prompting selection of the controlled crush injury paradigm to specifically assess treatment efficacy [43]. Post-operatively, 12 μL of the composite hydrogel was precisely administered to envelop the injury zone. Mild adhesion to adjacent tissues was observed by day 7, with partial biodegradation by day 14. By day 28, the hydrogel had nearly been resorbed, and the injured nerve displayed restored continuity with significantly reduced neuromuscular deficits (Fig. 4A upper). Functional recovery was evaluated via behavioral assays, demonstrating substantially improved outcomes in the BExos@GelMA/HAMA cohort. In this group, vibrissae movement symmetry and nasal deviation scores approached near-complete restoration (Fig. 4A lower). As depicted in Fig. 4B, total functional scores increased to 85 % of sham levels by day 28, surpassing the hydrogel-only (65 %) and injury-only groups (45 %) (*p* < 0.01). The regenerative process advanced rapidly within the initial 14 days, then plateaued subsequently. The hydrogel likely provides combined biomechanical scaffolding and biological signaling during the early regenerative phase, thereby critically enhancing axonal regeneration observed in later stages. At day 14, the sham group displayed normal neural transmission with an electromyography (EMG) latency of 1.23 ± 0.15 ms and conduction velocity of 5.4 ± 0.33 m/s. The injury group showed marked impairment, with latency prolonged to 3.88 ± 0.41 ms and velocity reduced to 0.9 ± 0.1 m/s. The BExos@GelMA/HAMA hydrogel group showed substantial recovery, achieving a latency of 1.89 ± 0.27 ms and a conduction velocity of 3.2 ± 0.4 m/s, outperforming both the injury and hydrogel-only groups (Fig. 4C). Amplitude recovery also reached 78.3 % by day 14 (*p* < 0.01). The electrophysiological data corroborated the behavioral findings, demonstrating that the composite hydrogel restored the fundamental electrophysiological properties for neural signaling.

Histochemical evaluations employing hematoxylin-eosin (H&E) and toluidine blue (TB) techniques demonstrated distinct morphological variations in cross-sectional specimens. Sham-operated specimens displayed orderly arranged axonal bundles with compact myelin sheaths and well-preserved Schwann cell arrangements. Conversely, injured nerves manifested characteristic Wallerian degeneration patterns featuring perineurial disruption, leukocyte accumulation, and extensive myelin loss. The GelMA/HAMA treatment cohort showed regenerative improvements including attenuated inflammatory responses, enhanced neural fiber integration, and partial myelin reconstruction. Notably, the BExos@GelMA/HAMA group approached histological normality with preserved neural microarchitecture, negligible immune cell infiltration (Fig. S8) and homogeneous myelin regeneration (Fig. 4D,E,F). The data collectively suggest that the hydrogel composite facilitates axonal regrowth and myelin restoration while mitigating inflammatory responses, thereby promoting functional neural recovery.

Quantitative PCR analysis provided molecular-level evidence (Fig. 4G). Compared with sham controls, the injury group showed significant downregulation NeuN, NF200, βIII-tubulin, and S100 (*p* < 0.001). In contrast, the composite hydrogel markedly restored these markers, with NeuN and NF200 expression reaching 85 % and 87 % of sham levels (*p* < 0.01), significantly higher than in the hydrogel-only group. Axonal structural genes (NF200, βIII-tubulin) exhibited particularly strong responses to exosome treatment (*p* < 0.01), suggesting exosomes preferentially enhance cytoskeletal reorganization during axonal regrowth. Fibrotic markers α-SMA and Collagen I were upregulated but decreased to 24.5 % and 19.8 % of injury levels following treatment (*p* < 0.001).These findings corroborate emerging evidence that exosomes regulate neural repair through RNA-mediated cellular reprogramming [[44], [45], [46], [47], [48]]. Western blot results confirmed the protein-level restoration achieved by BExos@GelMA/HAMA treatment. In the injury group, neural markers NeuN, NF200, and βIII-tubulin decreased, while fibrotic proteins α-SMA and Collagen I increased more than threefold, indicating severe axonal damage and fibrosis (Fig. 4H). The hydrogel-only group showed partial recovery. The Schwann cell marker S100, which is critical for myelination, showed complete normalization (92.3 ± 10.2 % of sham) in the composite group versus hydrogel alone (*p* < 0.01). These protein-level findings align with the observed electrophysiological improvements in conduction velocity. Notably, the key regulator of axonal growth cone dynamics βIII-tubulin showed particularly strong recovery (54.8 ± 8.2 %), suggesting the hydrogel's porous structure facilitates cytoskeletal reorganization. Concurrently, α-SMA and Collagen I dropped to about 20–25 % of injury values, confirming strong exosome-mediated antifibrotic activity [49].

### 2.4 Immunofluorescence analysis demonstrated the synergistic enhancement of BExos@GelMA/HAMA hydrogel in facial nerve repair within an extrusion injury model

**Fig. 5:**
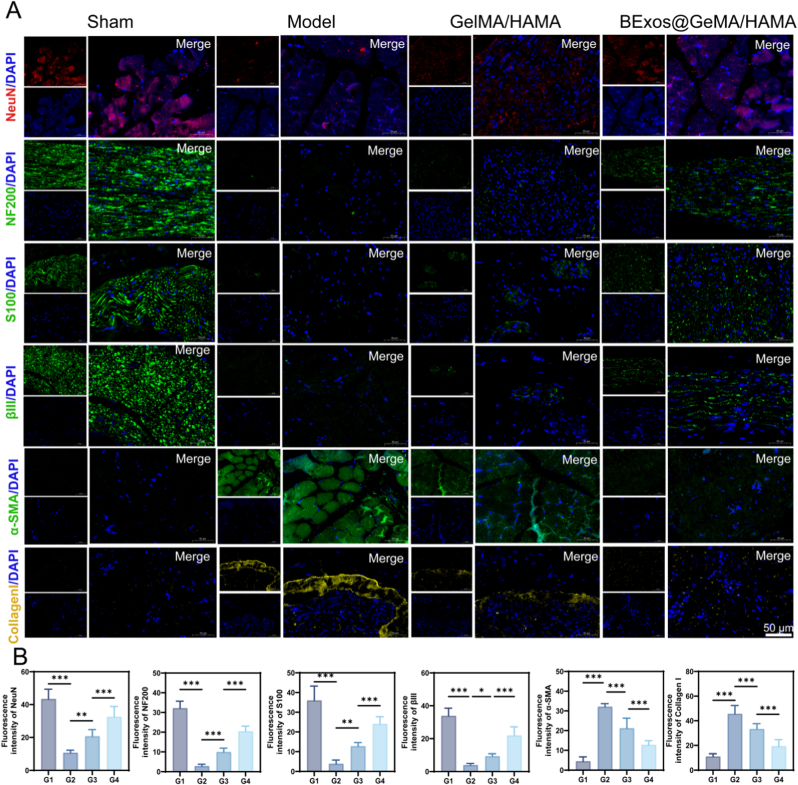
In vivo assessment of facial nerve repair and functional recovery. (A,B) Immunofluorescence staining. Scale bars: 50 μm. (G1: Sham. G2: PBS-treated. G3: GelMA/HAMA. G4: BExos@GelMA/HAMA. ∗*p <* 0.05; ∗∗*p <* 0.01; ∗∗∗*p <* 0.001.)

Immunofluorescence analysis revealed that BExos@GelMA/HAMA hydrogel intervention ameliorated the expression of these biomarkers, with the BExos@GelMA/HAMA hydrogel exhibiting superior efficacy in enhancing marker expression relative to standalone hydrogel therapy, suggesting exosome-mediated neuroregenerative effects (*p* < 0.001). Compared to the standalone hydrogel, the BExos@GelMA/HAMA hydrogel intervention improved the expression of biomarkers including NeuN, NF200, S100, and βIII, while reducing the expression of α-SMA and Collagen I. Fibrosis-associated proteins α-SMA and Collagen I showed substantial upregulation in injured specimens (>5 × sham group levels, *p* < 0.001), yet composite treatment attenuated these markers to approximately 40–45 % of those observed in the injury cohort, demonstrating enhanced therapeutic performance compared to hydrogel monotherapy (Fig. 5A and B). These findings collectively demonstrate the dual therapeutic efficacy of the BExos@GelMA/HAMA hydrogel, which not only enhances the expression of neural cell biomarkers but also maintains Schwann cell viability while effectively delaying fibrosis progression.

### 2.5 RNA-seq data analysis

**Fig. 6:**
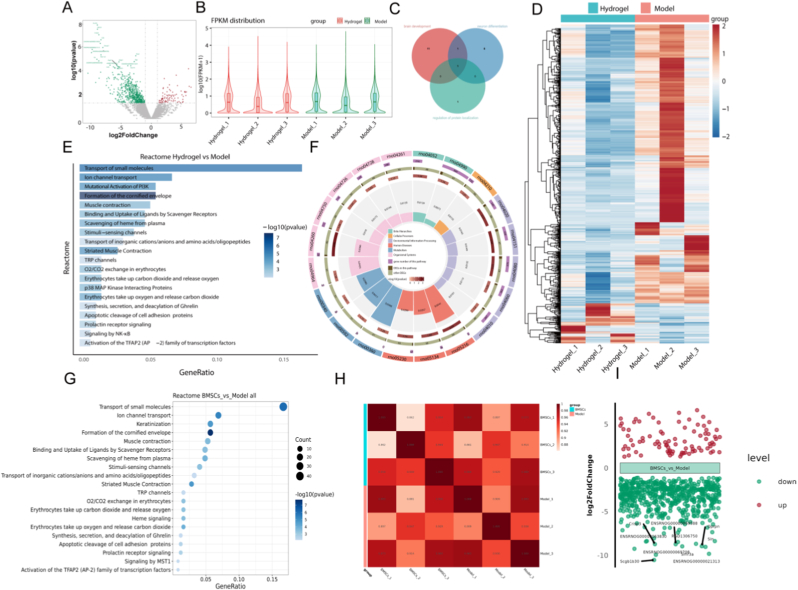
Bioinformatics analysis results. (A) Volcano plot of differentially expressed genes. (B) Violin plots of gene expression. (C) Venn diagram of pathway intersections. (D) Heatmap of 307 core downregulated genes. (E) Enriched pathway based on the *Nnat* expression. (F and G) Enrichment signaling pathways. (H) Sample correlation heatmap. (I) Differentially expressed gene set mapping across groups.

Transcriptomic analysis of facial nerve specimens through bulk RNA-seq demonstrated contrasting molecular signatures between control cohorts and BExos@GelMA/HAMA intervention groups, particularly regarding neural regeneration mechanisms. Our investigation of 19,851 genomic elements identified 685 with significant expression variations (86 demonstrating increased expression levels, while 599 displayed reduced activity) across experimental conditions (Fig. 6A). Violin plot validation confirmed consistent expression trends among biological replicates (Fig. 6B). Prioritization through adjusted p-value filtering revealed *Nnat* as the pivotal gene intersecting neurodevelopmental pathways, neural specification processes, and protein trafficking regulation (Fig. 6C). Pattern recognition analysis of 307 persistently suppressed genetic elements in hydrogel-treated specimens exposed synchronized transcriptional behavior (Fig. 6D). Functional annotation through Gene Ontology (GO) categorization associated these molecular regulators with apoptotic regulation, immune modulation, subcellular protein distribution, and synaptic adaptability (Fig. 6E), while Kyoto Encyclopedia of Genes and Genomes (KEGG) pathway mapping established connections between these genetic factors and p38, NK-κB and PI3K signaling regulation et al. (Fig. 6F and G). Using the expression levels (measured in RPKM or FPKM) for all genes in each sample, inter-group and intra-group sample correlations were computed and visualized through heatmap generation. This graphical representation effectively illustrates inter-group variations and intra-group experimental consistency. Elevated correlation values reflect greater similarity in transcriptional profiles among biological replicates. The sample correlation heatmap appears in Fig. 6H. Quantitative analysis of differentially expressed genes between groups is presented in Fig. 6I, with the x-axis denoting experimental groupings and the y-axis showing expression magnitude differences. Upregulated genes are marked in red and downregulated genes in green, with each group highlighting the ten most significantly altered genes. *Nnat* was identified as a shared gene involved in neural differentiation processes, cerebral maturation, and protein homeostasis pathways [50]. Previous analysis revealed high *Nnat* expression in neural progenitors and dynamic changes during the inflammatory-reparative transition, supporting its role in phenotype switching. These findings provided a molecular framework for understanding how BExos@GelMA/HAMA hydrogel promotes nerve regeneration by modulating transcriptional pathways.

### 2.6 Polarization phenotype of macrophages validation in vivo

**Fig. 7:**
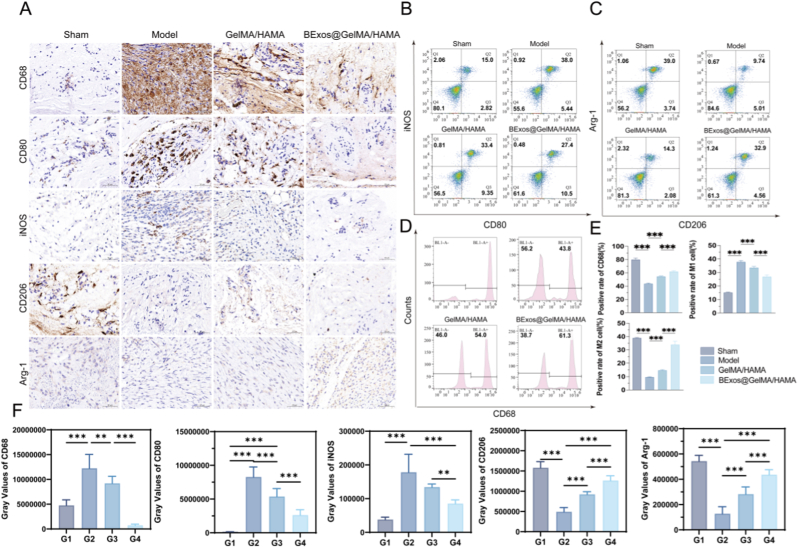
Polarization phenotype of macrophages validation in vivo. (A,F) Immunohistochemical staining of macrophage related proteins. Scale bars: 60 μm. (B,C) Stream-based detection of macrophage M1/M2 polarization markers. (D) Flow cytometry test of M0, M1 and M2 macrophage markers. (E). Quantitative results of macrophage detection by flow cytometry. (n = 5, G1: Sham. G2: PBS-treated. G3: GelMA/HAMA. G4: BExos@GelMA/HAMA. ∗*p <* 0.05; ∗∗*p <* 0.01; ∗∗∗*p <* 0.001.)

At 14 days post-injury, immunohistochemical analysis demonstrated the composite hydrogel's regulatory effects on macrophage dynamics following facial nerve trauma (Fig. 7A–F). Compared to sham-operated controls, injured specimens exhibited pronounced CD68^+^ cell accumulation within neural connective tissues (*p* < 0.001), reflecting robust inflammatory responses. Pro-inflammatory M1 markers (CD80^+^ and iNOS^+^) clustered predominantly in regions of axonal degeneration and leukocyte aggregation, while anti-inflammatory M2 indicators (CD206^+^ and Arg-1^+^) showed diminished presence restricted to regenerative niches and vascular interfaces. The results confirmed that M1 type was dominant in acute inflammation, and the hydrogel system could moderate the polarization of macrophages to M1 type and restore the polarization spectrum of macrophages to normal. Notably, composite hydrogel administration substantially downregulated CD80/iNOS expression while enhancing CD206/Arg-1 signals (*p* < 0.001), demonstrating the scaffold's capacity to rebalance macrophage phenotypes through microenvironment modulation demonstrating significant efficacy in regulating inflammatory responses and facilitating the differentiation of macrophages into the M2 phenotype. Macrophage polarization analysis revealed a marked M1 predominance in injured subjects (M1/M2 ratio = 6.8, *p* < 0.001). Composite hydrogel intervention demonstrated enhanced suppression of CD80 and iNOS expression compared to controls, effectively inhibiting pro-inflammatory pathway activation (*p* < 0.001, Fig. 7B). Immunofluorescence co-localization studies of CD206 and Arg-1 biomarkers verified complete phenotypic transition to M2 repair-oriented macrophages following composite hydrogel administration (Fig. 7C). Flow cytometric evaluation demonstrated diminished CD68^+^ macrophage populations in sham controls, reflecting attenuated immune responses. However, composite hydrogel therapy successfully normalized macrophage quantities, outperforming single-component hydrogel interventions (*p* < 0.001, Fig. 7D). Fig. 7E provides quantitative integration of polarization metrics from panels B–D. The composite formulation achieved near-normal polarization equilibrium (M1/M2 ratio = 0.6), exhibiting 39 % decreased pro-inflammatory CD80^+^/iNOS^+^ cells coupled with 2.3-fold elevated CD206^+^/Arg-1^+^ anti-inflammatory populations (*p* < 0.001). Comprehensive flow cytometric profiling systematically mapped temporal macrophage polarization patterns following facial nerve trauma and subsequent therapeutic interventions (Fig. S10). To validate the macrophage-mediated neuroprotective support, we further examined markers of Schwann cell differentiation at the cellular level using a co-culture model (RSC96 Schwann cells were cultured in 24-well plates 2 × 10^4^ cells per well and co-cultured with M2 RAW264.7 cell-conditioned medium for 48 h. In vitro co-culture experiments were performed after 24 h of incubation). As shown in Fig. S11, the results demonstrate that macrophages cultured with BExos@GelMA/HAMA effectively promote Schwann cell proliferation and differentiation.

### 2.7 BExos@GelMA/HAMA hydrogel may promote Nnat-mediated macrophage polarization through PI3K/NF-κB/P38 pathway modulation

**Fig. 8:**
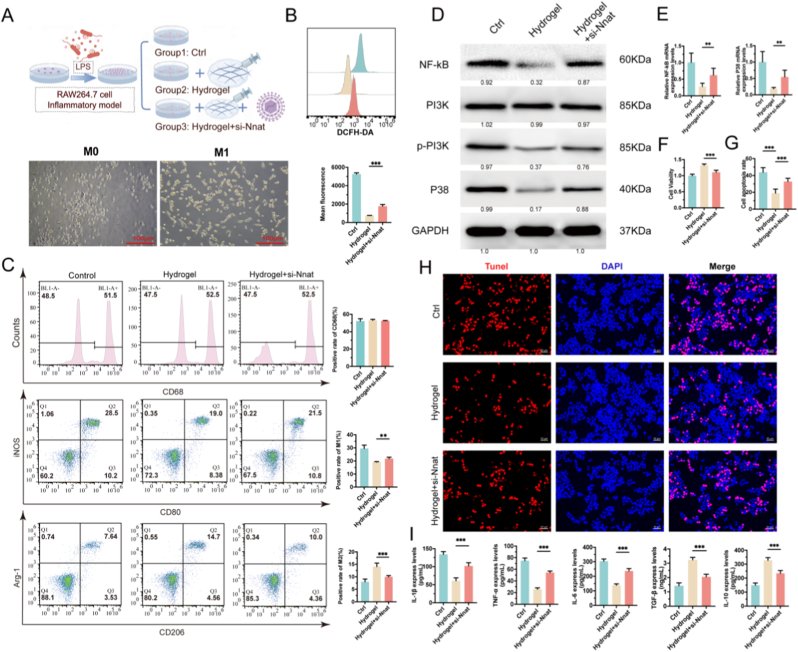
In vitro evaluation of immunomodulatory effects in composite hydrogel systems involving *Nnat*. (A) Top: Schematic representation of in vitro inflammatory model; Bottom: Morphological changes in macrophages observed via light microscopy. Scale bars: 100 μm. (B) Flow cytometric analysis of reactive oxygen species. (C) Phenotypic characterization of macrophage subtypes (M0/M1/M2) through surface marker detection. (D) Enhanced activation of NF-κB/P38 signaling pathways with elevated PI3K phosphorylation observed in Hydrogel + si-*Nnat* group versus hydrogel control. (E) Quantitative PCR analysis of gene expression. (F) Metabolic activity assessment through viability assays. (G) Apoptotic cell quantification. (H) TUNEL staining patterns across experimental groups. Scale bars: 50 μm. (I) Cytokine profile analysis using ELISA. (n = 5, ∗*p <* 0.05; ∗∗*p <* 0.01; ∗∗∗*p <* 0.001.)

Three groups were established in vitro, including the inflammation model (control), hydrogel intervention group, and hydrogel combined with *Nnat* gene knockdown group (Fig. 8A upper). Following LPS stimulation, RAW264.7 macrophages exhibited characteristic morphological alterations, such as pseudopodia extension and cell body contraction (Fig. 8A lower). Flow cytometry assessment of intracellular reactive oxygen species (ROS) served as a vital technique for elucidating the complex interplay between oxidative stress and macrophage polarization. Data demonstrated a positive correlation between elevated ROS levels and pro-inflammatory responses, with a slight increase in ROS in the si-*Nnat* group (*p* < 0.001, Fig. 8B). Additionally, no significant differences were observed in the proportion of CD68^+^ macrophages across groups, indicating that treatments did not influence macrophage viability (*p* > 0.05). Further evaluation of M1/M2 polarization markers revealed that the composite hydrogel facilitated M2 macrophage polarization, evidenced by decreased expression of iNOS and CD80, along with increased Arg-1 and CD206 levels. In the *Nnat* gene knockout group, this polarization phenomenon partially reversed, manifested as a significant increase in M1-type macrophages. This indicates that Nnat gene silencing shifts the polarization direction from M2-type to M1-type, thereby altering the inflammatory microenvironment (Fig. 8C). Western blot analysis indicated that *Nnat* silencing markedly mitigated the hydrogel's anti-inflammatory effects. Specifically, expression levels of NF-κB and P38 were significantly increased in the si-*Nnat* group compared to the hydrogel alone (*p* < 0.01). Phosphorylation of PI3K (p-PI3K) was also markedly elevated (*p* < 0.01), suggesting activation of inflammation-associated signaling pathways. These findings suggest that *Nnat* suppression amplifies PI3K pathway activation, subsequently stimulating downstream NF-κB and P38 signaling, thereby potentiating inflammatory responses. Disruption of the PI3K/Akt and NF-κB negative feedback mechanisms post-*Nnat* silencing potentially results in uncontrolled signal amplification and heightened inflammation (Fig. 8D). Quantitative PCR results supported these findings, showing increased expression of NF-κB and P38 in the *Nnat*-silenced group (Fig. 8E). CCK-8 assays demonstrated that hydrogel treatment (2 mg/mL) improved cell viability in LPS-activated macrophages. Nevertheless, the observed cellular protection was partly diminished through *Nnat* interference, as evidenced by decreased viability in the si-*Nnat* cohort relative to hydrogel-only controls (72.4 ± 3.9 %, *p* < 0.001), substantiating *Nnat* ‘s involvement in survival pathway regulation (Fig. 8F). Apoptosis analysis demonstrated that BExos@GelMA/HAMA hydrogel markedly suppressed macrophage cell death under inflammatory stress. While LPS-treated controls showed extensive apoptotic activity (38.6 ± 3.7 %), hydrogel application reduced this to 12.4 ± 2.1 % (*p* < 0.001, Fig. 8G). Genetic inhibition of *Nnat* partially restored apoptotic levels to 24.5 ± 3.3 % compared to hydrogel-treated samples (*p* < 0.001, Fig. 8H). Comparative fluorescence visualization revealed markedly fewer DNA fragmentation signals in hydrogel-treated macrophages versus LPS-challenged counterparts, suggesting the composite material's anti-apoptotic efficacy arises from synergistic interactions between its scaffold architecture and exosome-derived bioactive elements. The hydrogel's ability to improve cellular survival capacity and inhibit programmed cell death suggests that BExos@GelMA/HANA hydrogel establishes a biomimetic niche supporting macrophage viability in neural repair processes, where *Nnat* acts as a pivotal molecular modulator. LPS challenge triggered intense inflammatory activation, elevating IL-1β (3.8-fold), TNF-α (4.2-fold), and IL-6 (5.1-fold) concentrations relative to baseline measurements. Hydrogel administration substantially mitigated these pro-inflammatory responses, achieving 65–72 % reductions in pro-inflammatory markers while boosting anti-inflammatory IL-10 (2.9-fold) and TGF-β (3.4-fold) expression (*p* < 0.001). Interestingly, TGF-β, a pleiotropic cytokine participating in immune homeostasis and extracellular matrix remodeling, though its pro-fibrotic effects in pathological conditions warrant consideration [51]. This cytokine shift revealed the hydrogel's capacity to polarize macrophages toward immunoregulatory states. Genetic ablation of *Nnat* nullified these therapeutic outcomes, reinstating elevated IL-1β and TNF-α, IL-6 production while suppressing IL-10 secretion, highlighting the critical involvement of *Nnat* in hydrogel-driven immune modulation (Fig. 8I).

### 2.8 Immunofluorescence detection of inflammatory indicators of related pathways

**Fig. 9:**
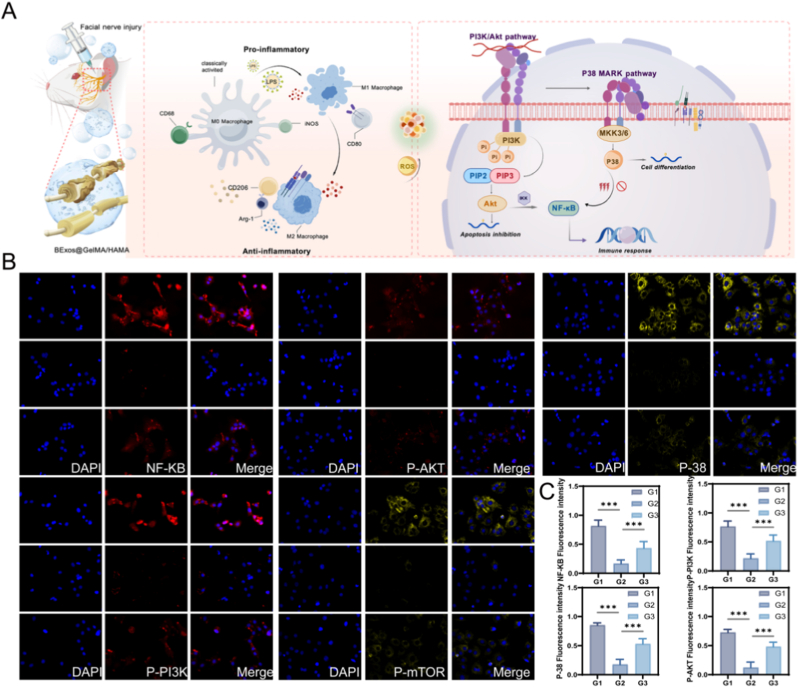
Immunofluorescence detection of Inflammatory indicators. (A) Schematic diagram of the changes in the expression of key genes in the inflammatory pathways in the composite hydrogel group. (B,C) mmunofluorescence staining. Scale bars: 50 μm. (n = 5, G1: Control group; G2: BExos@GelMA/HAMA hydrogel group; G3: BExos@GelMA/HAMA hydrogel + si-*Nnat* group. ∗*p <* 0.05; ∗∗*p <* 0.01; ∗∗∗*p <* 0.001.)

The BExos@GelMA/HAMA hydrogel suppresses the PI3K/Akt signaling cascade by preventing nuclear translocation of NF-κB. Following *Nnat* gene knockout, inflammatory pathways exhibit activation, potentially through amplified PI3K signaling that drives subsequent NF-κB and P38 stimulation (Fig. 9A) [[52], [53], [54]]. Expression profiles of NF-κB, P-PI3K, P-AKT-P, mTOR, and P38 were visualized through immunofluorescence analysis as demonstrated in Fig. 9B and C. Intriguingly, *Nnat* expression exhibited paradoxical upregulation post-knockdown. These findings establish a conceptual framework for developing intelligent targeted anti-inflammatory biomaterials. Comprehensive evaluation of the BExos@GelMA/HAMA system across diverse inflammatory models remains essential to maximize its translational value in biomedical contexts. Furthermore, we conclusively demonstrated that the therapeutic efficacy was primarily mediated by exosomes rather than solely by the hydrogel structure or residual soluble factors (Fig. S12). Additionally, we measured the levels of neurotrophic factors NGF and BDNF, both of which showed high expression. This further demonstrates that BExos@GelMA/HAMA promotes the maturation and differentiation of Schwann cells (Fig. S13).

### 2.9 Comprehensive biocompatibility and toxicological evaluation of GelMA/HAMA composite hydrogel

**Fig. 10:**
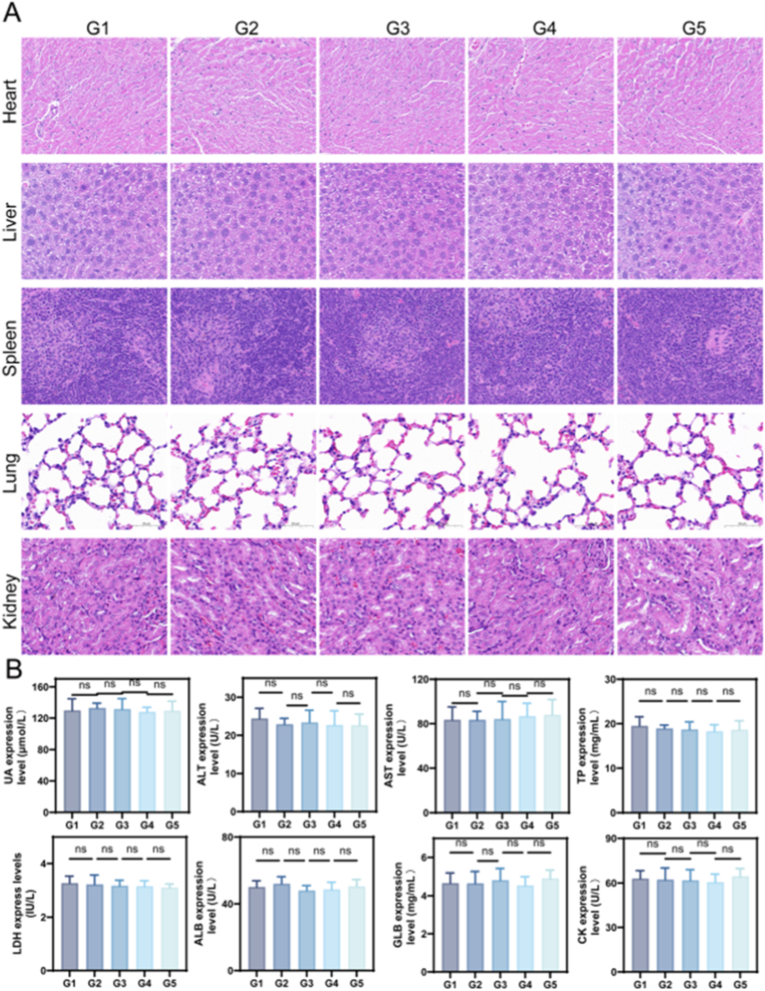
Analysis of biological safety in GelMA/HAMA Hydrogel. (A) H&E staining images of the main organs (heart, liver, spleen, lung and kidney the formation of various formulas. (B) Hemalum chemical analysis UA, ALT, AST, TP, LDH, ALB, GLB, CK. (n = 5, G1: PBS. G2: 10 %GelMA/0.5 %HAMA. G3: 10 %GelMA/1 %HAMA. G4: 10 %GelMA/1.5 %HAMA. G5: 10 %GelMA/2.5 %HAMA. ∗*p <* 0.05; ∗∗*p <* 0.01; ∗∗∗*p <* 0.001.)

Histological evaluations revealed comparable tissue integrity between hydrogel-treated specimens and PBS controls across vital organs including cardiac, hepatic, splenic, pulmonary, and renal tissues (Fig. 10A). The GelMA/HAMA composite system demonstrated outstanding biocompatibility in animal models, corroborating previous in vitro safety assessments. Post-euthanasia hematological profiling indicated stable hepatic enzyme levels and renal biomarkers within normal physiological ranges (Fig. 10B), with complete blood count parameters showing no statistically significant deviations from baseline measurements. This comprehensive toxicological evaluation confirms the material's suitability for biomedical applications, as both histological and serological analyses aligned closely with control group findings.

## 3 Methods

### 3.1 BExos@GelMA/HAMA preparation

**Preparation of GelMA/HAMA composite hydrogels.** GelMA/HAMA composite hydrogel fabrication. GelMA preparation involved methacrylation of gelatin (Macklin, China) using 8 % methacrylic anhydride in PBS solution at 50 °C with continuous agitation for 3 h. The resultant product underwent five-day purification through dialysis (12 kDa MWCO) followed by lyophilization [55]. For HAMA synthesis, hyaluronic acid (Macklin, China) was first dispersed in deionized water before undergoing 24-h methacrylation at pH 8.5, with subsequent termination through dialysis (12 kDa MWCO) and freeze-drying processes. The photoinitiator solution containing lithium phenyl-2,4,6-trimethylbenzoylphosphinate (LAP, Kemike, Wuhan, China) was formulated by dissolving 0.05 g LAP powder in 20 mL PBS, followed by thermal activation at 60 °C with periodic agitation for 15 min [56]. GelMA and HAMA were dissolved together in the LAP solution at three different Three distinct concentration ratios of GelMA and HAMA were co-dissolved in the LAP solution. This mixture was transferred into PDMS molds using pipettes and subjected to 405 nm ultraviolet irradiation. The hydrogel specimens were subjected to overnight freezing at −20 °C and subsequently examined using a field-emission scanning electron microscope (FE-SEM) operating at 2.0 kV acceleration voltage (Thermo Fisher Scientific, USA).

**NMR spectroscopy.** To confirm successful methacrylation and verify the chemical structures of GelMA and HAMA. Nuclear magnetic resonance characterization was conducted using an Inova-500M spectrometer (Varian, USA) to analyze the molecular structures of Gel, GelMA, HA, and HAMA. For analysis, 3–5 mg aliquots of each material were prepared in deuterium oxide (D_2_O) solvent until achieving optical clarity and uniform dissolution. Proton nuclear magnetic resonance (^1^H NMR) spectra were acquired at ambient temperature using the specified instrument. Spectral data interpretation and processing were executed through MestReNova analytical software.

**FT-IR spectroscopy.** Distinctive absorption bands were examined to verify the effective incorporation of methacrylate functional groups within both GelMA and HAMA structures. FT-IR analysis was conducted using approximately 3–5 mg of each specimen combined with dehydrated potassium bromide (KBr) at a 5 % mass proportion. The mixture was carefully blended and ground into a fine consistency using an agate mortar before being subjected to vacuum compression (20 mmHg pressure maintained for 5 min) to create transparent pellets. Spectral data collection was performed at ambient temperature using a Bruker VERTEX70 spectrometer (Germany) operating in transmission mode across the 4000-500 cm^−1^ spectral region. Distinctive absorption bands were examined to verify the effective incorporation of methacrylate functional groups within both GelMA and HAMA structures.

**Swelling ratio measurement.** To assess the equilibrium swelling behavior and determine how varying HAMA concentrations influence water absorption capacity and network structure stability. Equilibrium swelling characteristics were evaluated for hydrogels formulated with 10 % GelMA combined with varying HAMA concentrations (0.5–2.5 %). Specimens were fabricated into cylindrical shapes using 400 μL molds, followed by cross-linking process, removal from molds, and initial dry mass determination (W_dry_) employing a precision balance (BL610, Sartorius, Germany). The hydrated state analysis involved submerging samples in PBS at 37 °C, with subsequent surface moisture removal through gentle blotting using filter paper before recording the swollen mass (W_swollen_). Triplicate specimens were tested per experimental group, with mean values computed for data analysis. The equilibrium swelling capacity was determined using the following equation [57]:

Q (%) = [(W_swollen_ - W_dry)_ / W_dry_] × 100 %

**Compression measurement.** The mechanical resistance of hydrogel specimens under compressive forces was assessed through standardized testing protocols employing a universal materials analyzer [58]. Test specimens with standardized dimensions (height: 4.5 mm, diameter: 7.5 mm) underwent controlled deformation at predetermined strain rates until structural failure occurred. The testing apparatus continuously monitored the axial load (F) throughout the experiment. Compressive stress values (expressed in Pascals) were derived using the fundamental relationship:

Compressive Strength (Pa) = F / S

F is the applied compressive force (N) recorded at failure and S is the cross-sectional area (m^2^) of the hydrogel sample. Each group had five parallel samples, and the average value was calculated. Stress-strain curves were plotted. The elastic modulus was calculated as the slope of the stress-strain curve in a relatively uniform interval.

**Rheological mechanical property measurement.** The rheological behavior of composite hydrogels was analyzed using a Kinexus rotational rheometer (Malvern, UK) fitted with 25-mm-diameter parallel stainless-steel plates [59]. Experimental measurements were performed at ambient temperature conditions. To establish the linear viscoelastic regime, oscillatory frequency sweeps were implemented across 0.1–10 rad/s angular frequencies. Frequency-dependent measurements of storage modulus (G′) representing elastic behavior and loss modulus (G″) indicating viscous response were systematically recorded, enabling comprehensive evaluation of the materials' viscoelastic characteristics. The obtained modulus values served to distinguish between the solid-like and liquid-like components within the hydrogel network structure.

***In vitro* degradation ratio measurement.** To assess in vitro degradation characteristics, hydrogel specimens were equilibrated in PBS at 37 °C until reaching swelling equilibrium. Each 50 μL hydrogel aliquot underwent lyophilization to obtain baseline dry mass measurements (designated as W_0_). For enzymatic degradation simulation, samples were incubated in PBS supplemented with 1000 U/mL lysozyme solution. Following specified incubation periods, hydrogels were retrieved, carefully washed with ultrapure water, and subjected to secondary lyophilization. Post-treatment mass (Wt) was precisely measured after complete drying, with degradation percentage calculated through the established formula [60]:

Degradation rate (%) = (W_0_ - Wt)/ W_0_ × 100 %

**Coagulation Index Assays.** The hemocompatibility of lyophilized hydrogel specimens was evaluated using blood clotting index (BCI) measurements. Samples were thermally equilibrated at physiological temperature (37 °C) prior to testing. Experimental procedures involved sequential application of 100 μL citrate-treated whole blood and 20 μL of 0.2 M calcium chloride (CaCl_2_) solution onto each hydrogel surface. Following a 5-min incubation period, 25 mL deionized water was introduced to lyse residual erythrocytes through 5 min of controlled agitation. Optical density measurements were recorded at 545 nm wavelength using spectrophotometric analysis. The BCI values were determined using the equation [61]:

BCI (%) = (OD_sample / OD_negative control) × 100

**Hemolysis ratio test.** The hemolysis ratio test was conducted to evaluate the blood compatibility of experimental hydrogel formulations. Blood samples were collected from euthanized rats through cardiac puncture and immediately treated with heparin to prevent coagulation. Erythrocytes were separated through centrifugation (10,000×*g*, 5min) and subsequently subjected to three washing cycles using Dulbecco's phosphate-buffered saline (D-PBS). In the erythrocyte stability evaluation, 0.8 mL of prepared RBC suspension was combined with 200 μL aliquots of different solutions: 0.1 % Triton X-100 (positive control), PBS (negative control), and experimental hydrogel formulations. After 4-h incubation at ambient temperature, samples underwent centrifugation (10,000×*g*, 5min) and supernatant optical density was recorded at 540 nm using spectrophotometric analysis. Materials demonstrating hemolysis levels under 5 % were deemed biocompatible according to established criteria [62]. HR was calculated using the equation:

Hemolysis Ratio (%) = [(A - C) / (B - C)] × 100

**BExos isolation and preparation.** Following euthanasia and surface sterilization with ethanol, femurs and tibiae from adult male Sprague-Dawley rats (Cyagen, Suzhou, China) were surgically excised. Bone marrow-derived cells were harvested through aspiration, subjected to erythrocyte lysis, and subsequently cultured in high-glucose Dulbecco's modified Eagle medium. Cellular populations were maintained under standard conditions (37 °C, 5 % CO_2_), with non-adherent cellular components removed following a 48-h incubation period. Upon achieving 80–90 % confluency, BMSCs underwent PBS washing before transitioning to exosome-free culture medium. Exosome purification was conducted using a commercial isolation kit (BestBio, Shanghai, China), involving treatment with chilled isolation buffer (4 °C, 30 min) followed by sequential centrifugation (30 min, 4 °C). The purified exosome fraction was reconstituted in phosphate-buffered saline and cryopreserved at −20 °C for subsequent experimental procedures.

**Nanoparticle Tracking Analysis (NTA).** Hydrodynamic dimensions and size distribution profiles of the specimens were assessed via NTA employing HORIBA instrumentation. Experimental preparations involved adjusting sample concentrations with PBS to attain a 1 mL working volume. Dilution parameters were optimized to maintain 50-400 detectable particles per observation field, targeting ∼200 particles for enhanced tracking precision as referenced [63]. Thermal conditions during analysis were maintained at 24–26 °C using NanoSight's NTA software (v2.2) for particle trajectory monitoring. Experimental validation required triplicate analyses demonstrating particle count variations below 15 % coefficient of variation, consistent with established protocols [64].

**Transmission Electron Microscopy (TEM) observation.** After appropriate processing, the purified exosome samples were placed on a copper grid and observed using transmission electron microscopy (Thermo Fisher Scientific, USA).

**Three-dimensional immunofluorescence imaging.** Fluorescently labeled antibodies targeting specific exosome markers were incubated with exosomes. Subsequently, confocal microscopy (LEXT OLS5100 3D, Olympus, Japan) was used to perform three-dimensional imaging to observe the morphology and marker distribution of exosomes.

**Western blot assay.** The Western blot assay was performed to quantify and detect the evaluation of protein expression levels and the identification of changes in protein abundance. Following a 24-h incubation period, cultured cells underwent two rinses with ice-cold PBS before undergoing lysis in 0.5 mL RIPA buffer supplemented with 1 mM PMSF (ST506, Beyotime, China) under chilled conditions for 15 min. Protein quantification was performed through bicinchoninic acid (BCA, WB6501, NCM Biotech, China) colorimetric analysis, calibrated against a bovine serum albumin (BSA, Sigma-Aldrich, USA) reference standard. Electrophoretic transfer onto PVDF membranes (Beyotime, China) was executed at 300 mA constant current for 90 min, followed by overnight exposure to primary antibodies at 4 °C. Immunoreactive bands were detected using enhanced chemiluminescence (ECL) detection reagents, with subsequent densitometric analysis conducted through ImageJ software (National Institutes of Health, USA). Detailed antibody specifications appear in Supplementary Table 1.

**Quantitative real-time PCR (q-PCR).** Quantitative real-time PCR (q-PCR). Gene expression levels were assessed using q-PCR technology. Gene expression levels were assessed using q-PCR technology. Total RNA was extracted from rat facial nerve tissues using TRIzol reagent (Invitrogen, USA) following the manufacturer's protocol, and RNA purity and integrity were assessed by NanoDrop spectrophotometry and agarose gel electrophoresis. Reaction systems (20 μL volume) were assembled in RNase-free tubes, incorporating cDNA templates, paired forward/reverse primers (10 μM concentration per primer), Vazyme's 2 × Taq Pro Universal SYBR qPCR Master Mix, and molecular-grade water. All amplifications were conducted in triplicate using a real-time thermal cycler, with data analysis performed through the 2^–ΔΔCt^ quantification approach using housekeeping genes for normalization. Detailed primer nucleotide sequences are provided in Supplementary Table 2.

### 3.2 Biological function evaluation in vitro

**Immunofluorescence staining.** To evaluate the expression and spatial distribution of target proteins in organizational samples, tissue specimens were initially treated with 5 % BSA blocking solution (Sigma-Aldrich, USA) under ambient conditions. Tissue specimens were initially treated with 5 % BSA blocking solution (Sigma-Aldrich, USA) under ambient conditions. Primary antibodies were then applied at manufacturer-recommended concentrations and maintained at 4 °C overnight. Following three TBST rinses (Servicebio, Wuhan, China), fluorescent secondary antibodies were introduced for 60-min dark incubation at room temperature. After subsequent washing cycles, nuclear counterstaining was performed using DAPI. Specimens were preserved with Fluoroshield® mounting medium. Quantitative analysis of fluorescent signals was conducted through ImageJ software.

**CCK-8 assay.** To assess cellular viability and metabolic activity, actively dividing cells in exponential growth phase were cultured in 96-well plates with 6-h stabilization for proper adhesion. Bone marrow-derived mesenchymal stem cells (BMSCs) (Passage 2) were seeded onto the experimental substrates for adhesion, proliferation, and differentiation assays. Actively dividing cells in exponential growth phase were cultured in 96-well plates with 6-h stabilization for proper adhesion. CCK-8 reagent (10 μL/well) was introduced during the final 2 h of culture. Cellular metabolic activity was determined by measuring optical density at 450 nm using a microplate reader [65].

**Cell viability assay.** Assessment of cellular viability. Cellular viability evaluation was performed employing a dual-fluorescence viability detection kit (Calcein AM/EthD-I, Proteintech, China) [66]. Working solutions were prepared by diluting Calcein-AM (4 mM) and propidium iodide (1.5 mM) stock solutions under light-protected conditions. Actively proliferating cells underwent enzymatic detachment using trypsin, followed by two PBS rinses and resuspension in serum-free medium at concentrations ranging from 1 × 10^5^ to 10^6^ cells/mL. Cellular specimens were stained with 1 mL of the Calcein-AM/PI working solution through 37 °C incubation for 15–30 min in darkness. Fluorescence microscopic analysis was conducted using a 490 ± 10 nm excitation filter to visualize viable cells (green fluorescence) and necrotic cells (red fluorescence).

### 3.3 In vivo efficacy measurement

**Facial nerve crush injury model and behavioral analysis.** Experimental protocols received approval from the Animal Ethics Committee of Xinhua Hospital Affiliated to Shanghai Jiao Tong University School of Medicine (XHEC-F-2023-027). Sprague-Dawley rats (200–220 g, 6–8 weeks old) were randomly allocated into four experimental cohorts (n = 5 per group): (G1) sham-operated control, (G2) PBS-treated, (G3) GelMA/HAMA hydrogel, and (G4) BExos@GelMA/HAMA hydrogel. Surgical procedures were performed under aseptic conditions with combined anesthesia using ketamine (40 mg/kg) and xylazine (5 mg/kg). Following exposure of the right facial nerve trunk, a 2 mm segment adjacent to the stylomastoid foramen was subjected to three consecutive 60 s compressions with 1-min pauses using vascular clamp forceps. Local administration of 12 μL treatment solutions (PBS or hydrogel formulations) was performed peri-lesionally. Postoperative wound management included antiseptic treatment and layered closure. Neurological function evaluations occurred at postoperative days 1, 7, 14, 21, and 28, incorporating blink reflex testing through bilateral air stimulation (5 mL syringe, 3 cm ocular distance) as previously described [67]. Whisker mobility and nasal asymmetry were monitored over a 30 s interval. The composite evaluation score reflected the cumulative assessment of all criteria, where 0 denoted unimpaired physiological status while values exceeding 3 implied potential facial nerve impairment.

**H&E and TB staining.** Two weeks post-procedure, neural specimens encompassing the facial nerve and adjacent tissues (approximately 1 cm) were harvested, preserved in 10 % formalin, and prepared for myelin evaluation using dual staining techniques. Following a rinse and differentiation step with a 1 % acetic acid solution, tissue sections underwent sequential dehydration and were permanently mounted on slides (4 μm thickness). Microscopic examination and image documentation were performed at 400× magnification.

**Nerve conduction velocity measurement.** To assess nerve function recovery at multiple time points post-surgery, CMAP parameters were evaluated using a digital neurophysiological recording apparatus (ZY-J-Z100, Zhongke, Beijing, China) with bipolar stimulation. The in vivo electrophysiological measurements were performed on the 14th day after injury. CMAP parameters were evaluated using a digital neurophysiological recording apparatus (ZY-J-Z100, Zhongke, Beijing, China) with bipolar stimulation. Recording electrodes were positioned adjacent to the orbicularis oris musculature, while stimulation points were located at the junction between the buccal nerve branch and the vertical canthal axis. A subcutaneous reference electrode was implanted at the caudal region. Electrical stimulation parameters consisted of 0.2 ms square-wave pulses at 2.5 V intensity. Sequential evaluations of CMAP amplitude and onset latency were conducted across experimental groups at 7, 14, 21, and 28 days following surgical intervention.

**RNA-seq.** Transcriptomic sequencing was performed on facial nerve tissues between the composite hydrogel group and control group to analyze gene expression differences. The transcriptome was analyzed on the 14th day after injury. Transcriptomic profiling data were generated through microarray analysis of rat facial nerve injury specimens utilizing R statistical environment (v4.3.2). Probe identifiers were precisely aligned with gene nomenclature using AnnoProbe and tinyarray toolkits to develop genome-wide transcriptional profiles [68]. Dendrogram-based heatmap visualizations were created with pheatmap, while differential expression patterns were plotted through EnhancedVolcano. Statistically significant DEGs were selected based on thresholds of |log_2_FC| > 1.0 and adjusted *p*-values <0.05. Subsequent biofunctional characterization employed clusterProfiler for integrated multi-omics analysis, combining Gene Ontology annotation with KEGG pathway exploration to delineate molecular mechanisms [69], which revealed *Nnat* as the unique overlapping gene of interest.

**Immunohistochemistry.** To visualize and analyze the expression of target proteins in tissue sections, tissue sections were initially treated with a blocking buffer containing 5 % BSA, followed by incubation with primary antibodies at 4 °C for 16 h. Histological examination was performed on the 14th day after injury. Tissue sections were initially treated with a blocking buffer containing 5 % BSA, followed by incubation with primary antibodies at 4 °C for 16 h. After gradual temperature equilibration and three PBS rinses, specimens were exposed to HRP-conjugated secondary antibodies. Chromogenic visualization was achieved using DAB substrate (Sigma-Aldrich, USA), with subsequent nuclear counterstaining employing hematoxylin. Processed sections underwent sequential dehydration in graded ethanol solutions and clearing in xylene before brightfield microscopic examination.

**Flow cytometry.** Flow cytometry was utilized to quantify macrophage polarization and inflammatory status in injured tissues. Tissue specimens were dissected and enzymatically treated with a solution containing collagenase I (1 mg/mL) and DNase I (50 U/mL) in a temperature-controlled shaking bath maintained at 37 °C. The fluorescent probe was prepared by diluting stock solution 1:1000 in serum-free culture medium, achieving a working concentration of 10 μM. For cell surface marker detection, 50 μL aliquots of cellular suspension were mixed with 10 μL of fluorescently labeled antibodies targeting CD80, Arg-1, iNOS, and CD206 (Abcam, UK), followed by 60-min incubation under light-protected conditions. Following enzymatic dissociation through gentle pipetting, cells were resuspended in phosphate-buffered saline and subjected to two centrifugation cycles (1000 rpm, 5 min each). Intracellular reactive oxygen species were quantified using the DCFH-DA fluorescent indicator (Beyotime, China), with subsequent data processing conducted through FlowJo analytical software (version 10, Dako, USA).

**Collection of Blood and Tissue Samples.** At the study endpoint, animals were euthanized. Major organs (heart, liver, spleen, lungs, and kidneys) were collected and fixed in 4 % paraformaldehyde. Following paraffin embedding, sectioning, and hematoxylin and eosin (H&E) staining, histological integrity, inflammatory infiltration, and pathological alterations were evaluated using light microscopy. Concurrently, whole blood (2–3 mL) was collected via cardiac puncture into EP tubes and allowed to stand on ice for 30 min. Serum was subsequently separated by centrifugation at 3000 rpm for 15 min. A comprehensive assessment of systemic toxicity was performed by analyzing key serum biomarkers, including those for hepatocellular injury (alanine aminotransferase, ALT; aspartate aminotransferase, AST), hepatic synthetic function (albumin, ALB; total protein, TP), renal function (uric acid, UA), and muscle damage (creatine kinase, CK).

### 3.4 In vitro inflammation model establishment and verification

**RAW264.7 cell culture.** RAW264.7 (BH-C415, Bohui Biological Technology, China) macrophages were maintained in high-glucose DMEM medium supplemented with 10 % fetal bovine serum (FBS, Excell Bio, China) and 1 % penicillin-streptomycin solution (Beyotime, China), incubated under standard conditions (37 °C, 5 % CO_2_). For experimental procedures, cells were plated at 5 × 10^4^ cells/well in six-well plates and subsequently exposed to LPS (MlBio, Shanghai, China) over a 6-h period. Cellular activation was confirmed through microscopic observation of characteristic morphological alterations, including retraction of pseudopodia and diminished cell spreading. In the RAW264.7 macrophage inflammatory model, LPS was added together with the hydrogel at a final concentration of 200 ng/mL and incubated for 6 h to induce macrophage activation.

***Nnat* gene silencing.** *Nnat* gene silencing was achieved through siRNA targeting the NCBI reference sequence NM_001291128.1 (Supplementary Table 3). Transfection mixtures were prepared by mixing 2 μL of 20 μM siRNA with 200 μL serum-free medium, followed by incorporation of 10 μL RNAFit transfection reagent (HB-RF-1000, Hanbio, China), with vortexing for 10 s to ensure homogeneity. The prepared complexes were introduced into cultured cells within complete growth medium, establishing a final siRNA concentration of 20 nM for gene suppression experiments.

**TUNEL assay.** TUNEL assay aimed to detect and quantify apoptosis by identifying TUNEL-positive cells through fluorescent microscopy. Cellular samples were immobilized in 4 % paraformaldehyde solution for half an hour, followed by membrane permeabilization using 0.3 % Triton X-100 (Sigma-Aldrich, USA). The enzymatic reaction solution was formulated by mixing TdT enzyme with fluorescent labeling reagent in a 1:9 volumetric ratio. Aliquots of 100 μL from this preparation were dispensed into six-well culture plates and maintained at 37 °C under light-protected conditions for 60 min. Fluorescent microscopy quantification recorded TUNEL-positive cells displaying green emission, with apoptotic indices determined through normalization against DAPI-counterstained cellular populations [70].

**Enzyme-Linked Immunosorbent Assay (ELISA).** To measure cytokine expression and evaluate inflammatory responses, we coated 96-well microplates with protein-specific capture antibodies through overnight refrigeration at 4 °C. IL-1β (CB10205-Ra, COIBO BIO, CHINA), TNF-α (CB11057-Ra, COIBO BIO, CHINA), IL-6 (CB10218-Ra, COIBO BIO, CHINA), IL-10 (CB10194-Ra, COIBO BIO, CHINA), and TGF-β (CB11066-Ra, COIBO BIO, CHINA). We coated 96-well microplates with protein-specific capture antibodies through overnight refrigeration at 4 °C. After thorough washing cycles, sequential incubations involved application of detection antibodies and horseradish peroxidase-linked secondary antibodies (Abcam, UK). Chromogenic development proceeded for 15 min under 37 °C dark conditions before termination with 50 μL stopping reagent. Optical density measurements were recorded at 450 nm wavelength.

### 3.5 Statistical analysis

All experimental procedures were conducted in three independent replicates (n = 5). Results are expressed as mean values with standard deviations. Analytical procedures were carried out using GraphPad Prism version 10.0 (GraphPad Software, La Jolla, USA). Statistical comparisons between groups were evaluated through one-way analysis of variance supplemented by Tukey's multiple comparison test. The CCK-8 assay data underwent analysis using two-way ANOVA. A probability threshold of *p* < 0.05 was established for determining statistical significance.

## 4 Discussion

The BExos@GelMA/HAMA composite hydrogel system developed in this study demonstrates remarkable advantages and innovations in facial nerve repair. Compared to existing hydrogel-based therapeutic strategies, its core breakthrough lies in achieving deep synergy between “structural support” and “immunomodulation.” Traditional hydrogel scaffolds, such as collagen-based neural conduits, primarily provide physical guidance but exhibit limited biological activity and often suboptimal repair effects in inflammatory environments. The GelMA/HAMA hydrogel in this study not only offers superior mechanical properties and controllable degradation rates but, more importantly, serves as an exosome delivery vehicle that actively reshapes the immune microenvironment following injury. By polarizing macrophages from pro-inflammatory M1 to anti-inflammatory M2, this system effectively inhibits chronic inflammation and secondary fibrosis, creating an ideal biological environment for neural regeneration. This strategy that integrates biomaterials with the essence of cell therapy transcends the limitations of single-functional scaffolds, representing a conceptual shift from “passive support” to “active regulation.” In terms of innovation, this study provides profound insights into its molecular mechanisms, which many existing researches have not achieved. Through transcriptomic analysis, we have for the first time identified Nnat as a key gene mediating hydrogel-induced immunomodulation and elucidated its specific pathway of regulating macrophage phenotype transformation via the PI3K/NF-κB/P38 signaling pathway. This discovery not only provides robust molecular evidence for the hydrogel system's efficacy, but also paves the way for developing intelligent neural repair materials targeting specific gene targets. Furthermore, using BMSC-derived exosomes instead of live cells in transplantation effectively circumvents challenges like immune rejection, low survival rates, and ethical concerns. The hydrogel's sustained-release mechanism also addresses the exosome's short half-life limitation, significantly enhancing both safety and therapeutic efficacy.

## 5 Conclusion

In conclusion, our experimental findings revealed that the BExos@GelMA/HAMA hydrogel system significantly enhanced facial nerve regeneration and functional restoration in animal models. Functionally, the hydrogel composite served dual purposes: establishing a three-dimensional framework supporting Schwann cell attachment and expansion while simultaneously regulating the immunological milieu through macrophage phenotype switching from M1 to M2 subtypes. This biological mechanism appeared partially mediated through the PI3K/NF-κB/P38 molecular axis, with *Nnat* emerging as a crucial regulator of cellular survival pathways. The hydrogel formulation potentially regulates macrophage behavior via redox-sensitive mechanisms, as evidenced by reduced reactive oxygen species generation. Additionally, the biomaterial system demonstrated capacity to minimize fibrotic tissue formation and collagen accumulation, consequently improving axonal myelination and neural signal transmission. These collective results underscore the therapeutic promise of exosome-encapsulated photo-crosslinkable hydrogel systems as an innovative regenerative approach for peripheral nerve regeneration while elucidating the underlying mechanisms governing their immunomodulatory functions and neural repair capabilities.

## CRediT authorship contribution statement

**Chun Chen:** Writing – original draft, Visualization, Validation, Methodology, Investigation. **Yifei Zhang:** Methodology, Formal analysis. **Linchao Zhang:** Software, Formal analysis, Data curation. **Israr Ullah:** Software, Data curation. **Lei Hang:** Validation, Software, Methodology, Formal analysis. **Yupeng Liu:** Writing – review & editing, Project administration, Formal analysis, Data curation. **Jun Yang:** Visualization, Resources, Project administration, Funding acquisition, Conceptualization.

## Ethics approval and consent to participate

Animal research protocols received prior authorization from the Animal Experimental Ethics Committee at Xinhua Hospital, affiliated with Shanghai Jiao Tong University School of Medicine (Approval No. XHEC-F-2023-027), and strictly adhered to the guidelines outlined in the National Institutes of Health's manual for laboratory animal welfare and experimental practices.

## Declaration of competing interest

We declare that we have no financial and personal relationships with other people or organizations that can inappropriately influence our work, there is no professional or other personal interest of any nature or kind in any product, service or company that could be construed as influencing the position or the review of the manuscript.
