# Supplementary material for: Exosome-functionalized photocrosslinked GelMA/HAMA hydrogel promotes facial nerve recovery via inflammatory microenvironment regulation
Source: Bioact Mater. 2026 Jan 19;60:1–19. doi: 10.1016/j.bioactmat.2026.01.008 (PMC12856441; doi:10.1016/j.bioactmat.2026.01.008)
Supplement: Multimedia component 1 [file mmc1.docx]

Supporting information

For

Exosome-Functionalized Photocrosslinked GelMA/HAMA Hydrogel Promotes Facial Nerve Recovery via Inflammatory Microenvironment Regulation

Fig. S1. 10% GelMA/2.5%HAMA hydrogel scanning electron microscopy images.


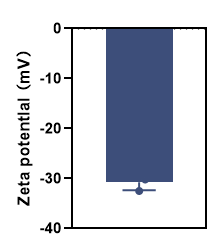


Fig. S2. Zeta potential data of BMSCs-Exo.


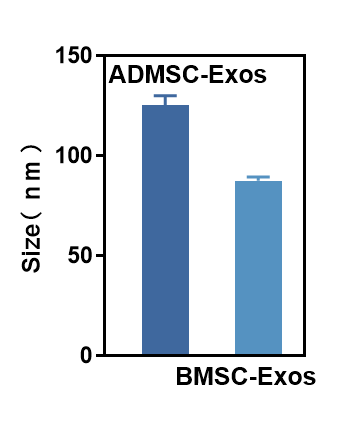


Fig. S3. Size data.


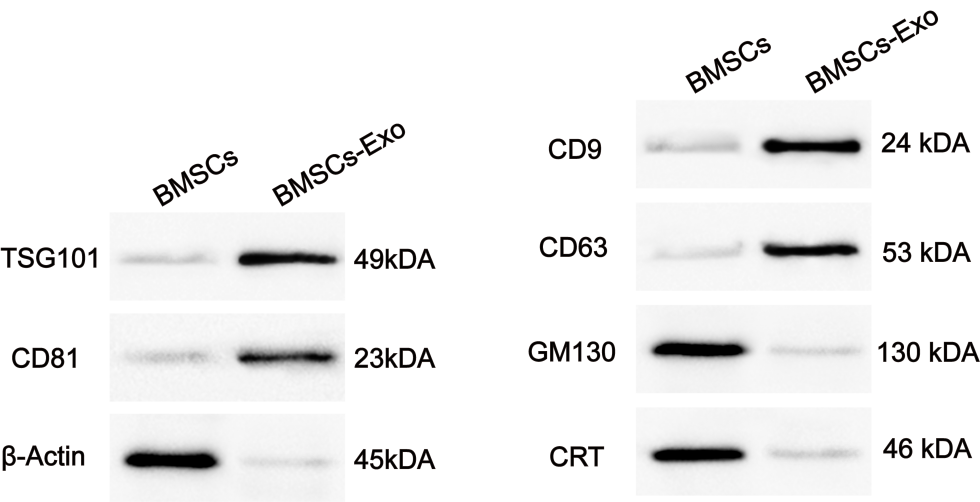


Fig. S4. WB detects exosome positive and negative markers.


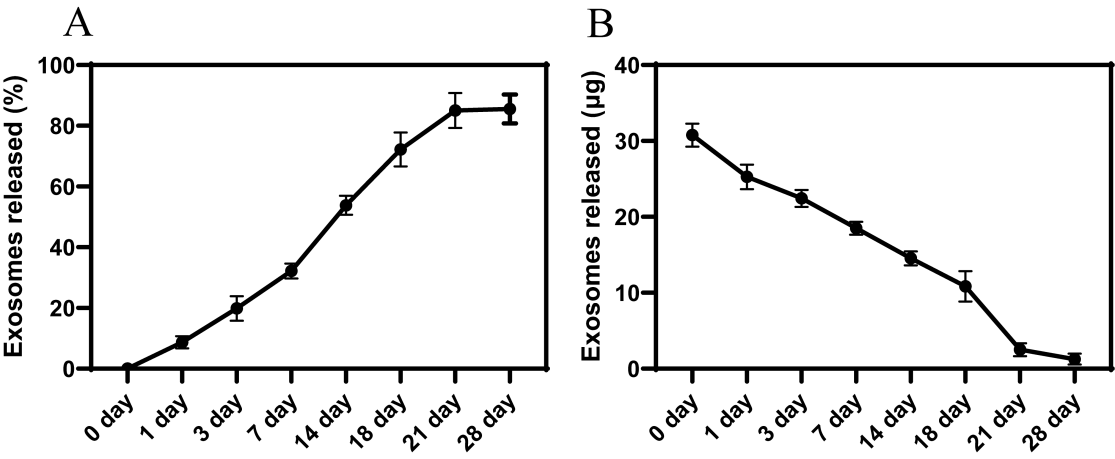


Fig. S5. (A) Cumulative release of BExos from BExos@GelMA/HAMA hydrogel over a 14-day period. (B) Daily BExos release profile from BExos@GelMA/HAMA hydrogel.


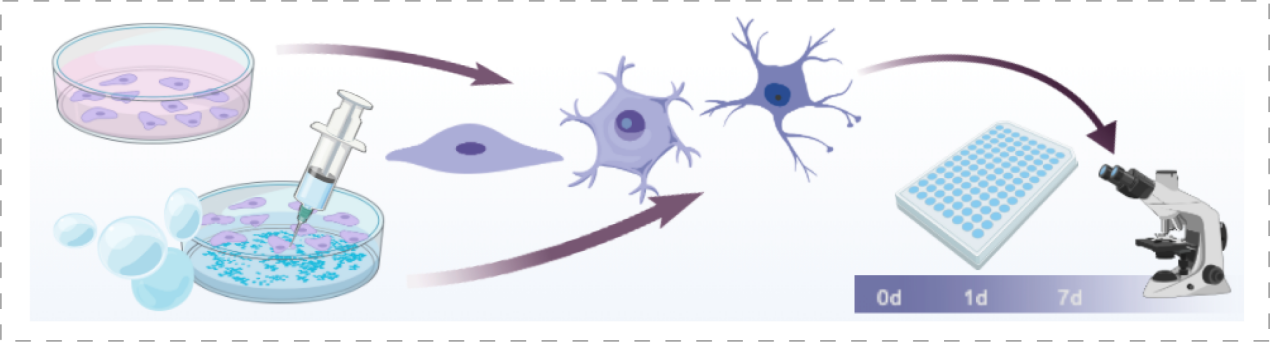


Fig. S6. Schematic diagram of in vitro cell experiment.

Fig. S7. (A) Macrophage morphology. (B-C) q-PCR analysis of S100β and SOX10 mRNA expression. (D) Quantitative analysis of diffusion area. (E) Quantitative analysis of synaptic length. (**p* < 0.05; ***p* < 0.01; ****p* < 0.001.)


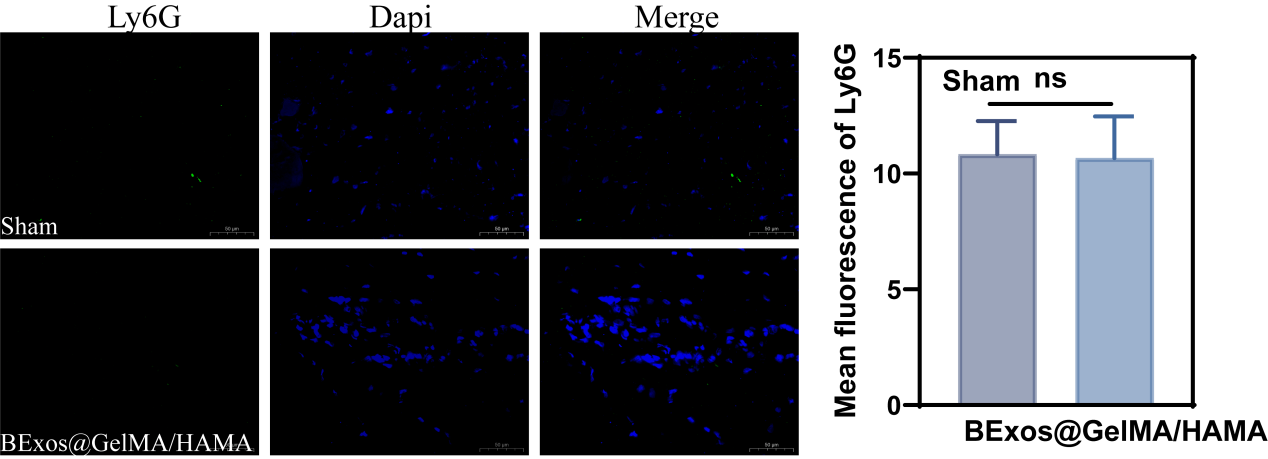


Fig. S8. Expression of neutrophils in facial nerve by immunofluorescence.(**p* < 0.05; ***p* < 0.01; ****p* < 0.001.)


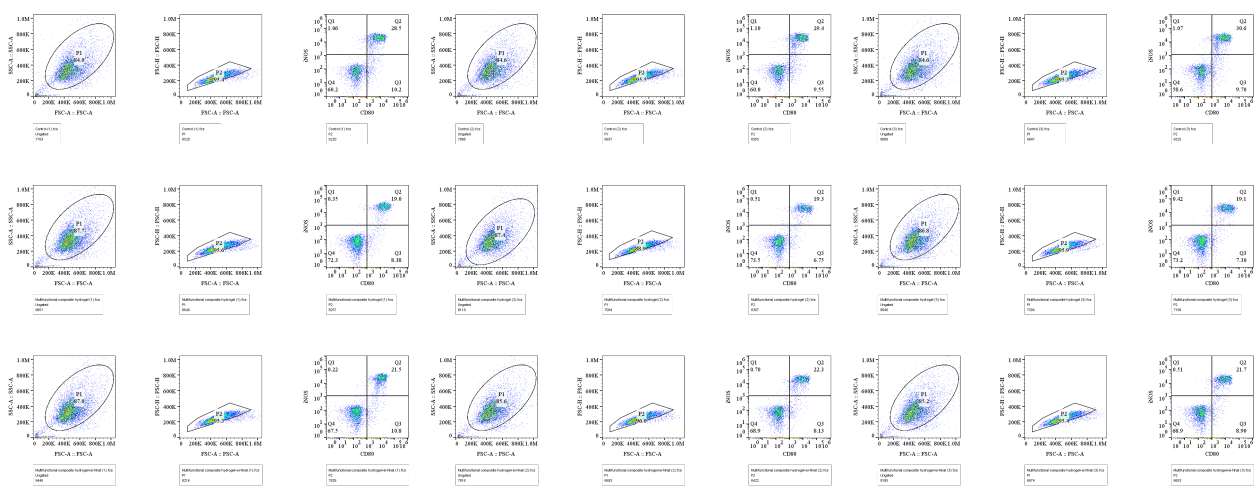


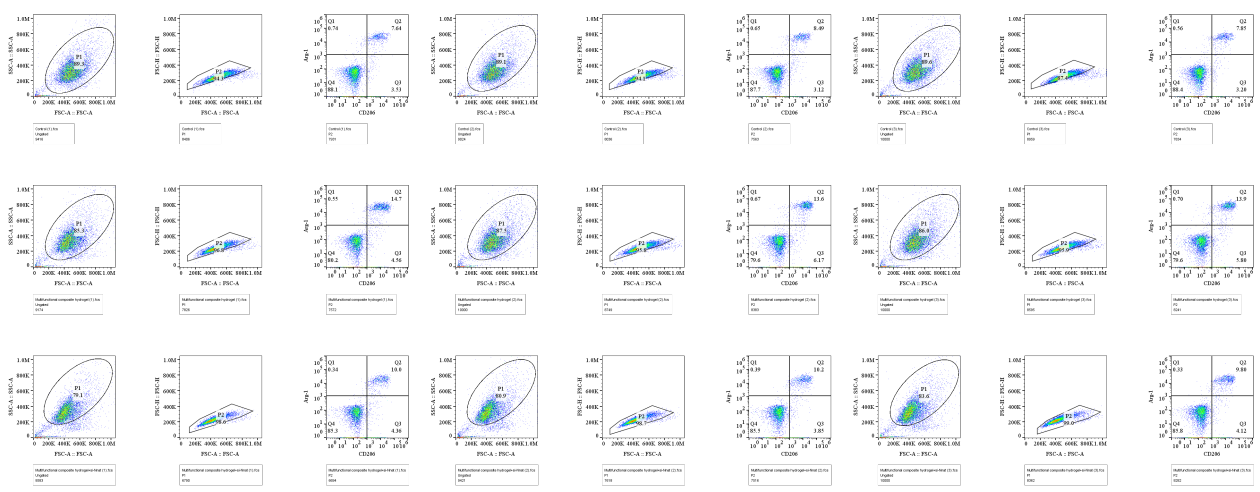


Fig. S9. Gate strategy of macrophage in flow cytometry.


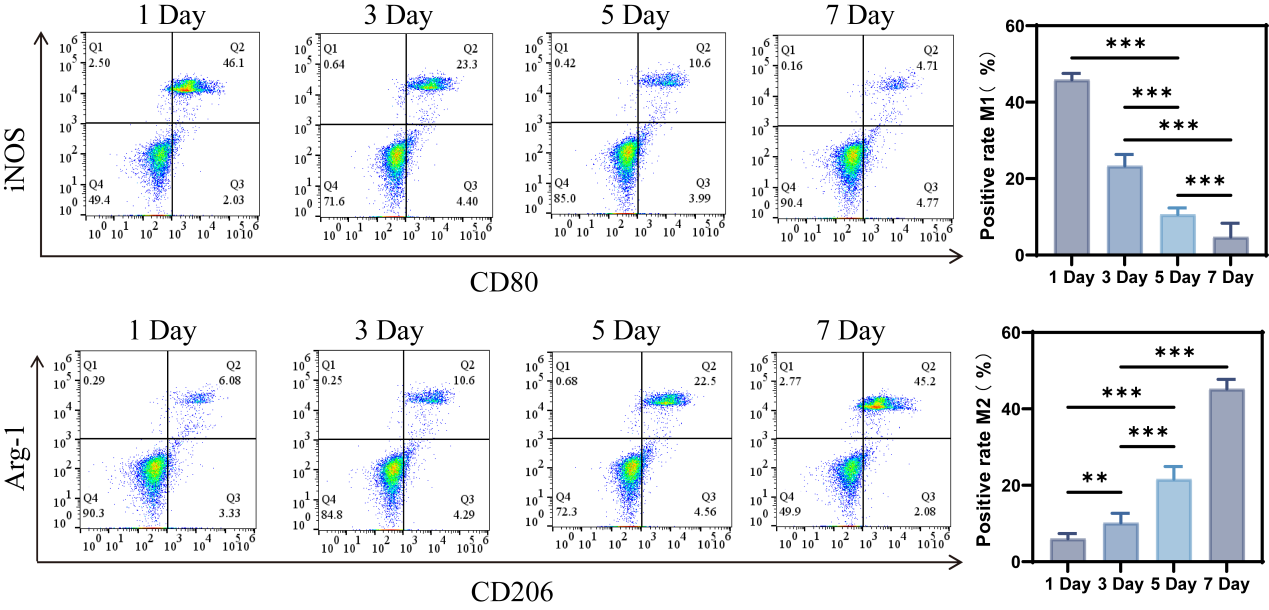


Fig. S10. Flow cytometry analysis of macrophage polarization at different time points.(**p* < 0.05; ***p* < 0.01; ****p* < 0.001.)


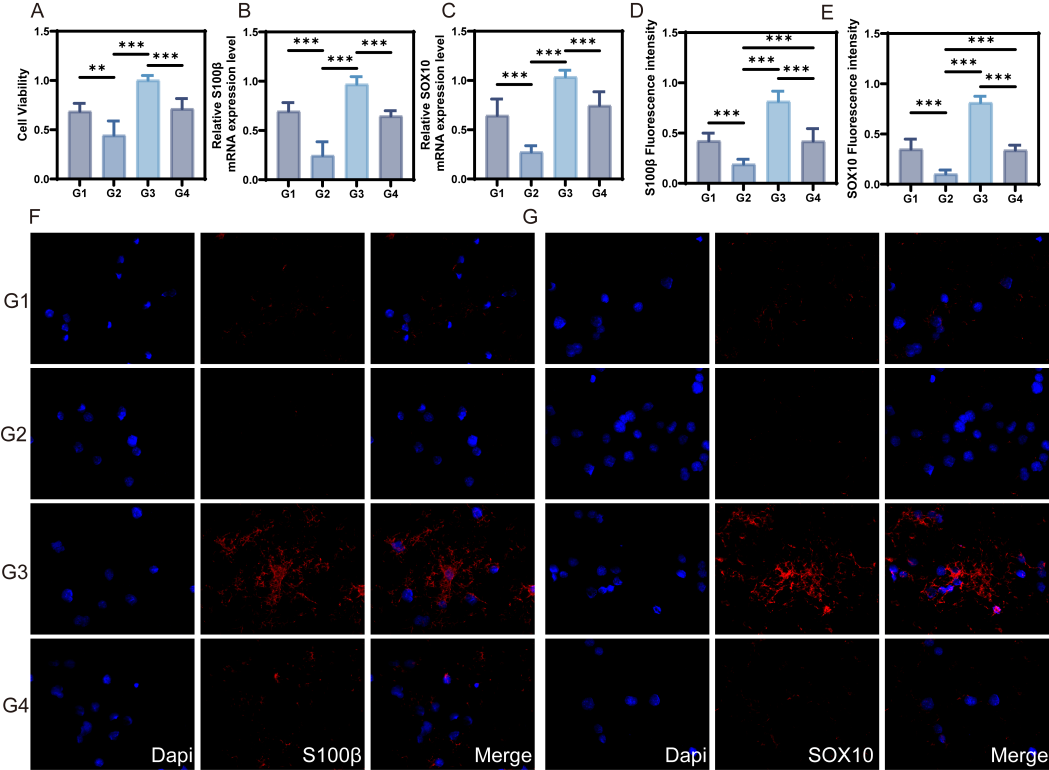


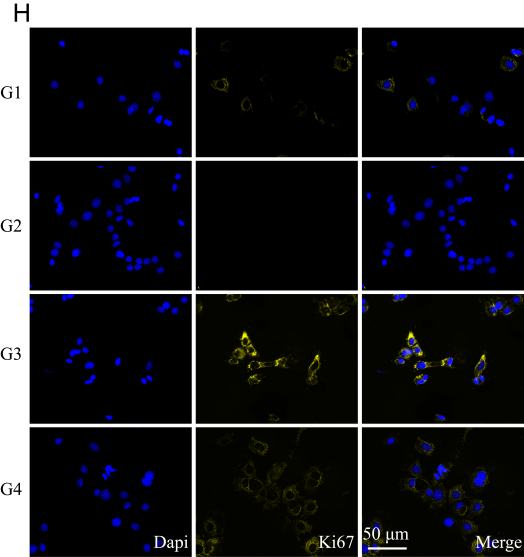


Fig. S11. (A) CCK-8 assay demonstrates cell proliferation. (B-C) q-PCR quantifies the mRNA expression of S100β and SOX10. (D-G) Immunofluorescence analysis reveals the protein expression of S100β and SOX10. (H) Immunofluorescence analysis reveals the protein expression of Ki67. (**p* < 0.05; ***p* < 0.01; ****p* < 0.001.)


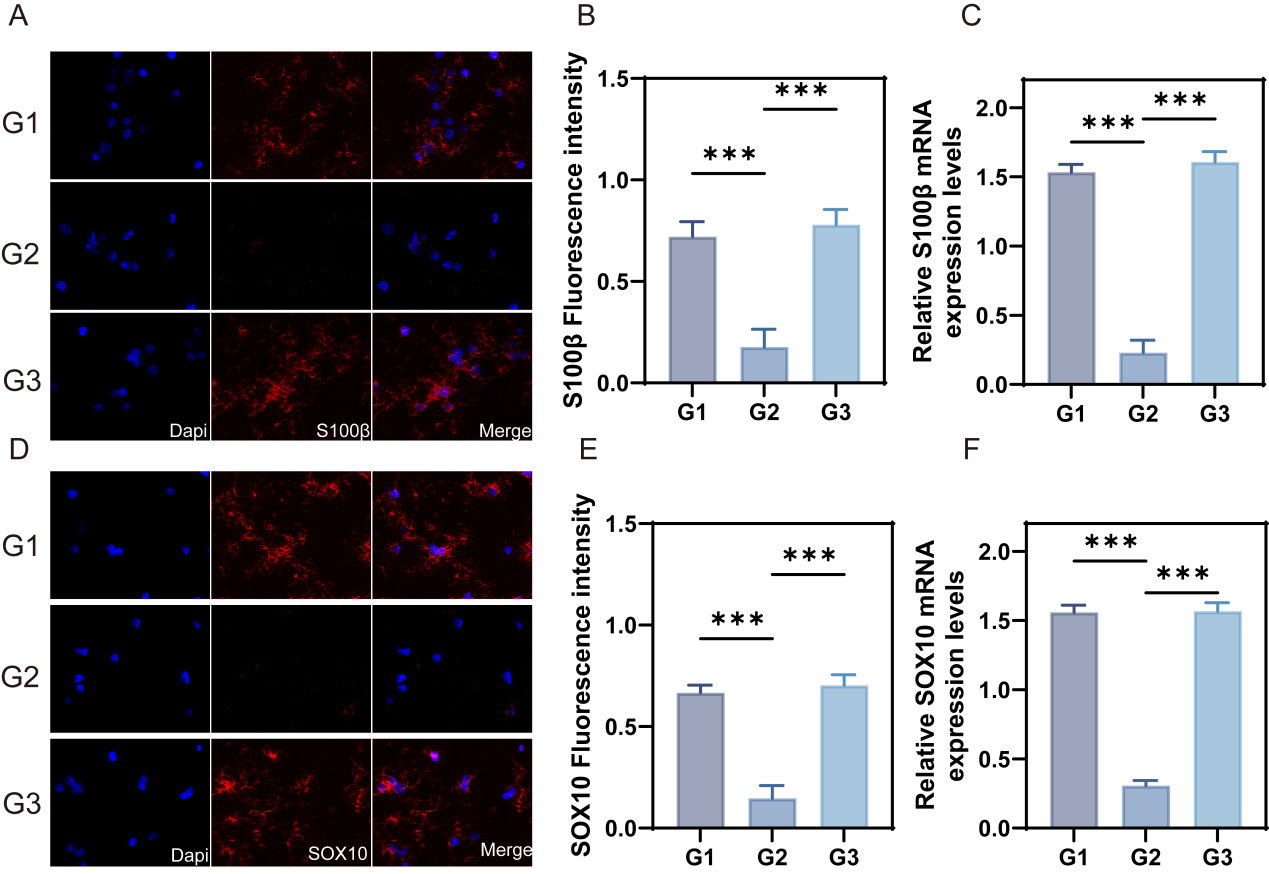


Fig. S12. (A-C) shows the expression of S100β detected by immunofluorescence (IF) and quantitative polymerase chain reaction (qPCR). (D-F) displays the expression of SOX10 detected by IF and qPCR. (**p* < 0.05; ***p* < 0.01; ****p* < 0.001.).


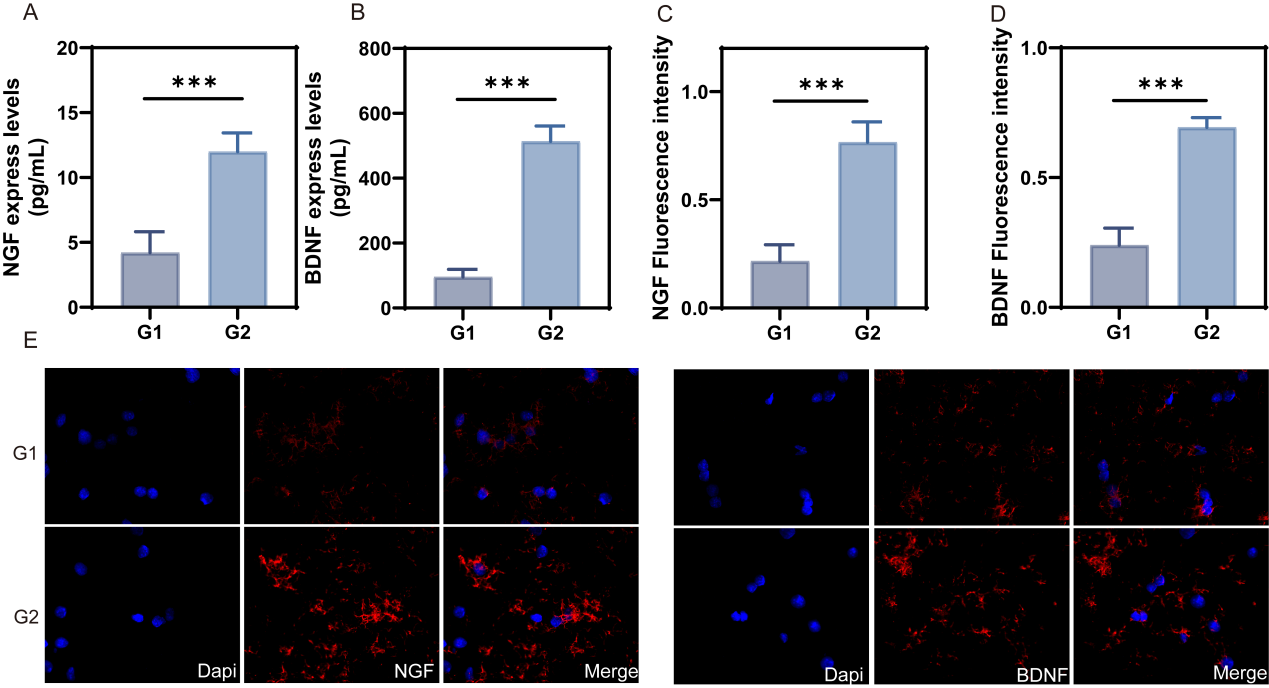


Fig. S13. (A-B) Elisa Expression of NGF and BDNF was detected by kit. Fig. (C-E) Expression of NGF and BDNF was detected by immunofluorescence. (**p* < 0.05; ***p* < 0.01; ****p* < 0.001.).
