# Supplementary material for: Exosome-functionalized photocrosslinked GelMA/HAMA hydrogel promotes facial nerve recovery via inflammatory microenvironment regulation
Source: Bioact Mater. 2026 Jan 19;60:1–19. doi: 10.1016/j.bioactmat.2026.01.008 (PMC12856441; doi:10.1016/j.bioactmat.2026.01.008)
Supplement: Multimedia component 2 [file mmc2.docx]

**Supplementary Table 1.** **Antibody dilution ratio**

| Antibody | Brand | Type | Dilution multiple |
| --- | --- | --- | --- |
| CD81 | Invitrogen | MA5-32333 | 1:1000 |
| TSG101 | Invitrogen | MA1-23296 | 1:1000 |
| CD9 | Invitrogen | MA5-31980 | 1:1000 |
| CD63 | Invitrogen | PA5-100713 | 1:1000 |
| GM130 | Invitrogen | MA5-47668 | 1:500 |
| CRT | Invitrogen | MA5-32131 | 1:5000 |
| Ly6G | Invitrogen | MA5-51254 | 1:10000 |
| NeuN | Abcam | ab104224 | 1:500 |
| βIII Tubulin | Abcam | ab18207 | 1:1000 |
| NF200 | Sigma-Aldrich | N4142 | 1:500 |
| S100 | Invitrogen | MA5-12969 | 1:500 |
| α-SMA | Sigma-Aldrich | A2547 | 1:1000 |
| Collagen I | Invitrogen | PA5-95137 | 1:1000 |
| GAPDH | Sigma-Aldrich | [G8795](https://www.sigmaaldrich.cn/CN/zh/product/sigma/g8795) | 1:10000 |
| Actin | Invitrogen | MA1-06110 | 1:10000 |
| NFκB p65 | Abcam | ab76302 | 1:1000 |
| PI3K p85 alpha | Invitrogen | MA1-74183 | 1:1000 |
| Phospho-PI3K p85 alpha | Invitrogen | PA5-104853 | 1:1000 |
| p38 MAPK | Abcam | ab31828 | 1:1000 |
| Goat Anti-Rabbit IgG  H&L(HRP) | Bioss | bs-0295G-HRP | 1:20000 |
| Goat Anti-Mouse IgG  H&L(HRP) | Bioss | bs-0296G-HRP | 1:20000 |

**Supplementary Table 2. Primer sequence**

| Primer |  | Sequence (5’→3’) |
| --- | --- | --- |

| S100β | Forward | ATGGAGACGCTGGACGAAGA |
| --- | --- | --- |
|  | Reverse | AGGCTGTGGTCACCATGGA |
| SOX10 | Forward | AATCCACCCGAAGCTAGAGG |
|  | Reverse | GATGACAAGTTCCCCGTGTG |
| β-actin | Forward | CTGGGACGACATGGAGAAAA |
|  | Reverse | AAGGAAGGCTGGAAGAGTGC |
| NeuN | Forward | CATGACCCTCTACACGCCAG |
|  | Reverse | GTCTGTGCTGCTTCATCTGC |
| βIII | Forward | TCCGAGTACCAGCAGTACCA |
|  | Reverse | GGCTTCCGATTCCTCGTCAT |
| NF200 | Forward | GAGCTGCTCGGTCAGATCCA |
|  | Reverse | GTCCAACCTCACTCGGAACC |
| S100 | Forward | CCTGGATGTCCAGAAGGATGC |
|  | Reverse | CCACTTCCCCATCTCCGTTTT |
| α-SMA | Forward | GGAGATGGCGTGACTCACAA |
|  | Reverse | CGCTCAGCAGTAGTCACGAA |
| Collagen I | Forward | GGAGAGAGCATGACCGATGG |
|  | Reverse | GGGACTTCTTGAGGTTGCCA |
| P38 | Forward | ACCTAAAGCCCAGCAACCT |
|  | Reverse | GTCATTTCGTCATCAGTGTGC |
| NF-κB | Forward | GTTGAGGGGACTTTCCCAGGC |
|  | Reverse | TCAACTCCCCTGAAAGGGTCCG |
| GAPDH | Forward | GGTCCCAGCTTAGGTTCATCA |
|  | Reverse | AATCCGTTCACACCGACCTT |

**Supplementary Table 3. *Nnat* siRNA sequence**

| *Nnat* siRNA-1 |
| --- |
| 5’-UGUACCAGCCGAUGAUGAGCA-3’  3’-CUCAUCAUCGGCUGGUACAUC-5’ |

| *Nnat* siRNA-2 |
| --- |
| 5’-AGCUUCUGCAGGGAGUACCUG-3’  3’-GGUACUCCCUGCAGAAGCUGG-5’ |

| *Nnat* siRNA-3 |
| --- |
| 5’-AUUCCUGCGCGGCACUGGCUC-3’  3’-GCCAGUGCCGCGCAGGAAUGG-5’ |

| si-NC |
| --- |
| 5'-UUCUCCGAACGUGUCACGUTT-3'  5'-ACGUGACACGUUCGGAGAATT-3' |
